# Generative artificial intelligence creates delicious, sustainable, and nutritious burgers

**DOI:** 10.1038/s41538-026-00953-x

**Published:** 2026-06-26

**Authors:** Vahidullah Tac, Christopher D. Gardner, Ellen Kuhl

**Affiliations:** 1 Department of Mechanical Engineering, Stanford University, Stanford, USA; 2 Prevention Research Center, Stanford University School of Medicine, Stanford, USA

**Keywords:** Mathematics and computing, Science, technology and society

## Abstract

Food choices shape both human and planetary health; yet, designing foods that are delicious, nutritious, and sustainable remains challenging. Here we show that generative artificial intelligence can learn the structure of the human palate directly from large-scale, human-generated recipe data to create novel foods within a structured design space. Using burgers as a model system, the generative AI rediscovers the classic Big Mac without explicit supervision and generates novel burgers optimized for deliciousness, sustainability, or nutrition. Compared to the Big Mac, its delicious burgers score the same or better in overall liking, flavor, and texture in a blinded sensory evaluation conducted in a restaurant setting with 101 participants; its mushroom burger achieves an environmental impact score more than an order of magnitude lower; and its bean burger attains nearly twice the nutritional score. Together, these results establish generative AI as a quantitative framework for learning human taste and navigating complex trade-offs in principled food design.

Food choices rank among the most consequential decisions humans make, with far-reaching implications for both personal and planetary health^1^. The global food system contributes substantially to climate change^2^, drives land-use change and biodiversity loss^3^, depletes freshwater resources^4^, and pollutes terrestrial and aquatic ecosystems^5^. Sustaining the Earth within a safe operating space for humanity requires a rapid transition toward more sustainable food systems^6^.

The same system exerts a dominant influence on human health^7^. More than one billion people consume diets that fail to meet basic nutritional needs^8^, while many more experience micronutrient deficiencies^9^. Poor dietary patterns contribute substantially to the global burden of chronic non-communicable diseases^10^, including type II diabetes^11^ cardiovascular disease^12^, and certain cancers^13^. Diet thus links environmental sustainability and human health through shared consumption patterns^14^.

Consumer acceptance, rather than availability, remains the central bottleneck in the adoption of sustainable and nutritious foods^15^. Deficits in taste^16^, texture^17^, and cultural familiarity^18^ continue to limit uptake, even when environmental and nutritional benefits are clear^19^. Designing foods that satisfy environmental and nutritional objectives, while meeting sensory expectations, demands a quantitative understanding of the human palate^20^. Yet, conventional food development relies heavily on artisanal expertise and incremental trial-and-error, which constrains systematic exploration of large design spaces and slows innovation^21^.

To address this gap, we leverage generative artificial intelligence to model human food preferences as a high-dimensional probability distribution, with burgers as a model system^22^. Rather than encoding rules for flavor or texture, the model learns statistical regularities directly from ingredient combinations and quantities. Unlike transformer-based large language models^23^ that generate recipes through next-token prediction in natural language^24^, our custom-designed diffusion model learns a structured probability distribution over ingredient identities and quantities. This probabilistic formulation enables quantitative sampling, controllable novelty, recipe rediscovery, and explicit optimization with respect to environmental impact and nutritional quality, while remaining grounded in consumer-relevant designs. While large language models excel at generating culinary instructions^25^ and broad world knowledge^26^, diffusion-based generative models provide greater control over ingredient compositions and probabilistic exploration of structured food design spaces. *We test the hypothesis that generative artificial intelligence can learn the human palate and create burgers that taste the same or better than the classic Big Mac®.*

The Big Mac® ranks among the most widely consumed burgers worldwide^27^, it is sold in more than 100 countries^28^, and its price serves as a widely used informal indicator of purchasing power parity across currencies^29^. This global familiarity makes the Big Mac® a stringent benchmark to evaluate whether a generative model captures collective human taste preferences. To test this premise, we evaluate the model using two discovery benchmarks: the rediscovery of the Big Mac® from statistical structure alone and the creation of novel burgers that retain high consumer acceptance.

Beyond validation, we integrate environmental sustainability and nutritional quality as additional criteria to select from a large ensemble of generated recipes. We quantify environmental impact using land use, greenhouse gas emissions, eutrophication potential, and scarcity-weighted water use^30^, and assess nutritional quality using established profiling frameworks, including the healthy eating index^31^. By sampling broadly and selecting recipes that jointly satisfy palatability, sustainability, and nutrition, we show that generative artificial intelligence can identify burgers that substantially reduce environmental impact and improve nutritional quality without abandoning cultural familiarity. Sensory validation with more than 100 participants confirms that the model correctly captures human taste preferences and creates burger recipes that match or exceed the sensory appeal of the classic Big Mac®.

## Results

### Generative AI successfully creates burger recipes

**Fig. 1:**
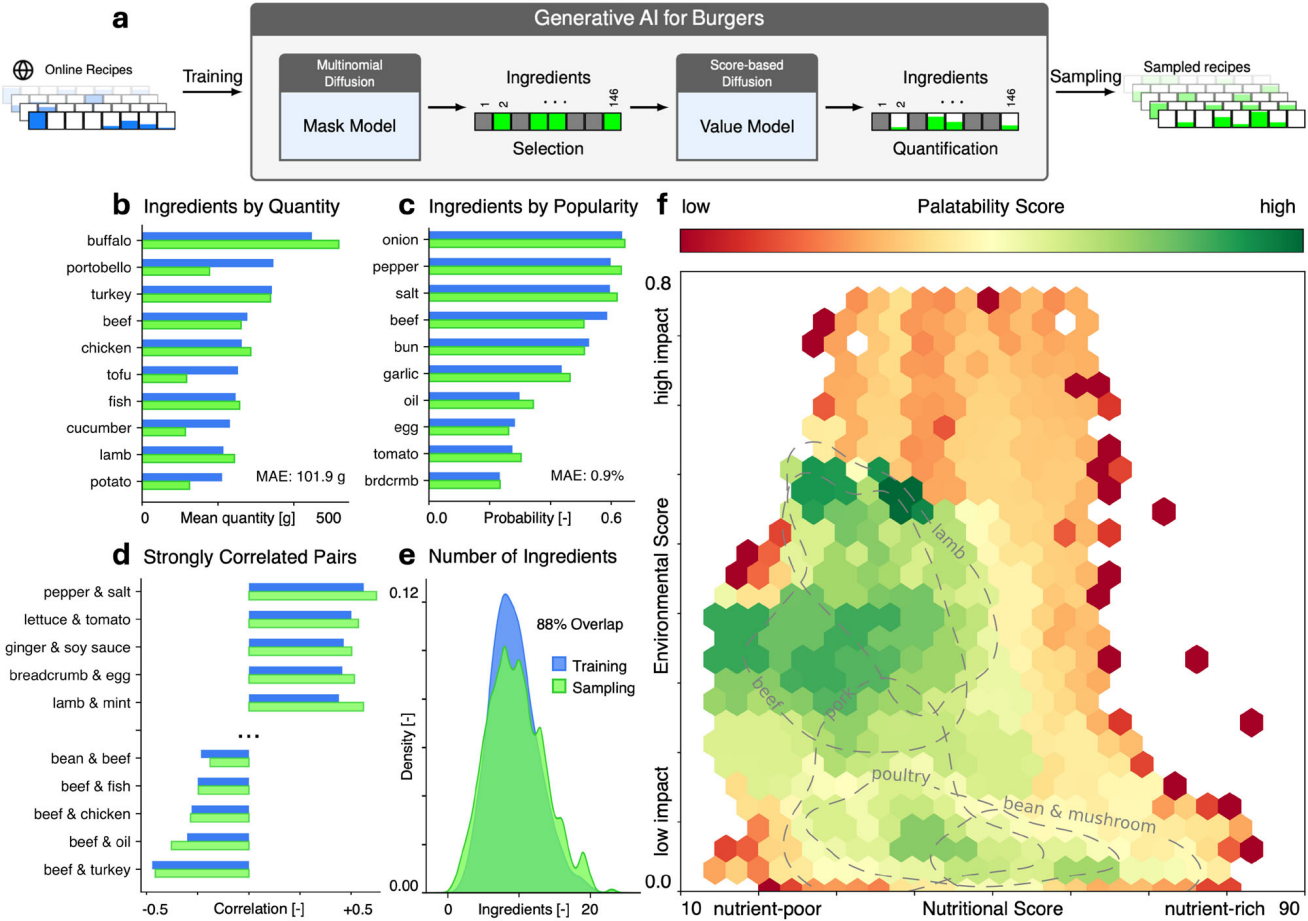
Generative AI for Burgers. High-level model architecture with multinomial diffusion model for ingredient selection and score-based diffusion model for ingredient quantification (**a**); comparison of training and sampling data in terms of ingredients by quantity (**b**) and popularity (**c**); strongly correlated pairs (**d**); and total number of ingredients (**e**); distribution of palatability score, environmental score, and nutritional score in 1 million samples where a high palatability (green) indicates a popular ingredient combination and a low palatability (red) indicates a rare ingredient combination (**f**).

We first validate the generative AI model by testing whether it reproduces key statistical properties of 2216 human-designed burger recipes while generating novel combinations (Fig. 1). The model architecture combines a multinomial diffusion model for ingredient selection with a score-based generative model for ingredient quantification, which together generate complete burger recipes defined by 146 ingredients and their quantities (Fig. 1a). Comparison of generated samples with the training data shows close agreement across multiple marginal and higher-order statistics: In particular, the model reproduces the distributions of ingredient quantities (Fig. 1b) and ingredient popularity, defined as the probability of ingredient occurrence across recipes (Fig. 1c), which demonstrates that it learns both how often ingredients appear and in what amounts. The model also captures higher-order structure, including strong positive and negative correlations between ingredient pairs commonly observed in real recipes (Fig. 1d), and accurately matches the distribution of recipe length, measured by the total number of ingredients per burger (Fig. 1e). After establishing statistical fidelity, we generate one million burger recipes and map their palatability, environmental, and nutritional scores to reveal the structure of the generated design space (Fig. 1f). Recipes with high palatability scores cluster in regions associated with popular, conventional ingredient combinations with lower nutritional and medium environmental scores, whereas recipes with low palatability scores occupy regions associated with rare or unconventional combinations, consistent with human culinary preferences. Together, these results demonstrate that the generative model learns the underlying distribution of real human-designed burger recipes and enables systematic exploration of trade-offs between palatability, nutrition, and environmental impact.

### Generative AI rediscovers classic burgers and creates novel designs

**Fig. 2:**
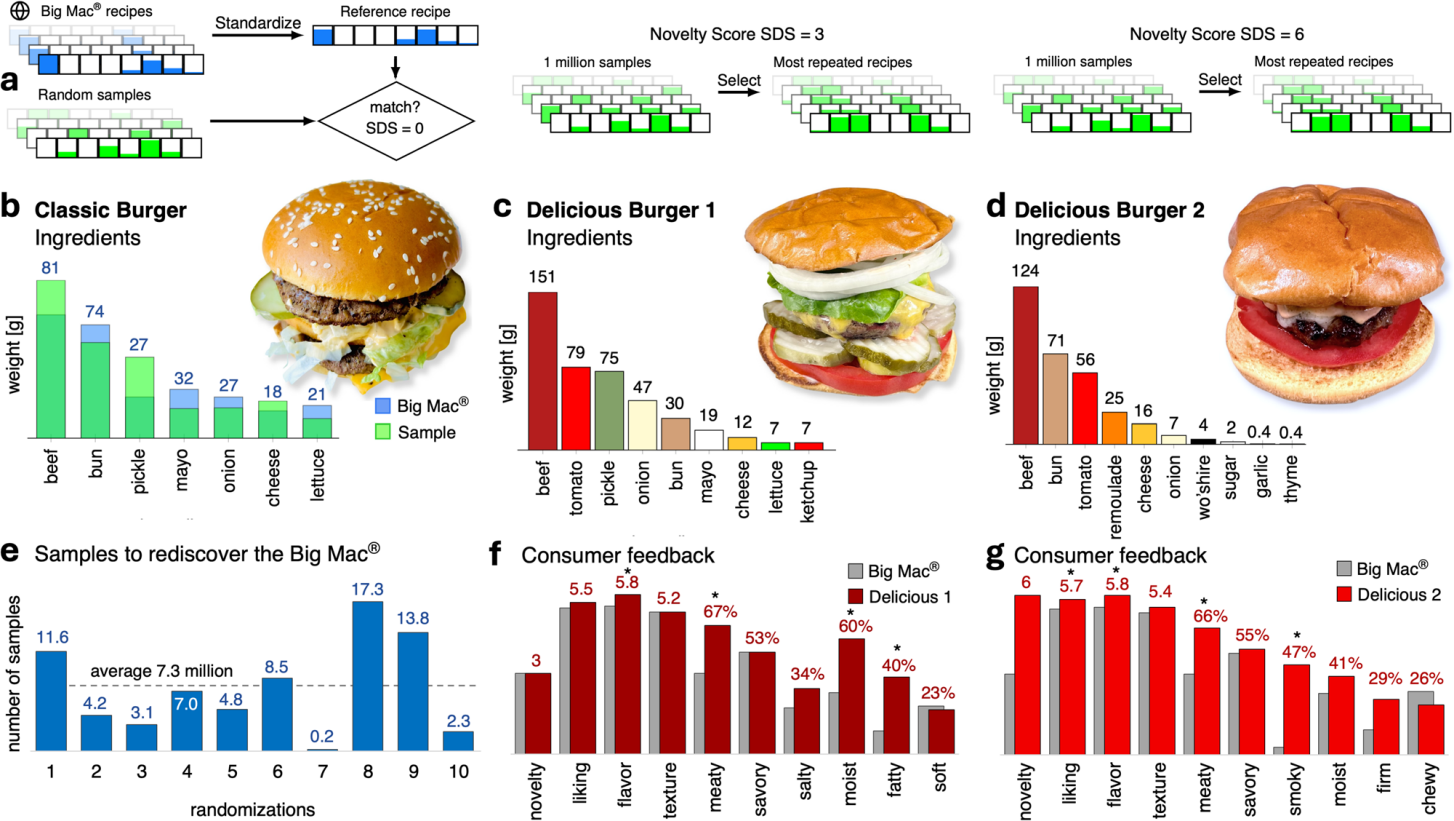
Rediscovering Classic Burger and Discovering of Delicious Burgers. The generative AI rediscovers classic burgers and discovers novel burgers from random AI generated samples, where a substantial difference score of zero, SDS = 0, defines a match between a classic recipe and the sample, and a substantial difference score larger than zero, SDS > 0, defines the novelty score of the sample (**a**); rediscovered recipe of the Big Mac® as representative Classic Burger (**b**); discovered recipes of Delicious Burger 1 with SDS > 3 and Delicious Burger 2 with SDS > 6 (**c**, **d**); number of samples needed to rediscover the Big Mac® for *n* = 10 randomizations (**e**); consumer feedback for Delicious Burger 1 and Delicious Burger 2 compared to the Big Mac® (**f**, **g**); * indicates statistical significance (*p* < 0.05).

We next assess whether the generative AI model can both rediscover canonical burger recipes and generate novel, appealing alternatives (Fig. 2). We quantify similarity between generated samples and a reference recipe using a substantial difference score, where SDS = 0 indicates a match in ingredients and quantities, and SDS > 0 measures increasing novelty (Fig. 2a). From random samples, the model successfully rediscovers the classic Big Mac®, both in correct ingredients and weights, although the Big Mac® was never part of the initial training data (Fig. 2a). Across ten independent randomizations, rediscovering the Big Mac® requires on average 7.3 million samples which demonstrates that exact replication of recipes is a low-probability event under the learned distribution (Fig. 2e). Beyond rediscovery, the model generates new burgers with varying degrees of novelty, illustrated by two representative recipes, the Delicious Burger 1 with SDS = 3 and the Delicious Burger 2 with SDS = 6, which exhibit progressively more distinct ingredient profiles while retaining familiar burger structure (Fig. 2c, d). Sensory evaluation indicates that the two delicious burgers achieve consumer ratings that are comparable to, and in some cases exceed, those of the classic Big Mac® (Figs. 2f, g): Delicious Burger 1 received significantly higher ratings than the Big Mac® for flavor (5.8 ± 1.3 vs. 5.4 ± 1.5) and Delicious Burger 2 for overall liking (5.7 ± 1.2 vs. 5.3 ± 1.5) and flavor (5.8 ± 1.3 vs. 5.4 ± 1.5), whereas both texture ratings did not differ significantly from the Big Mac® (*n* = 101, *p* < 0.05). Participants more frequently described the Delicious Burger 1 as meaty (67% vs. 42%), moist (60% vs. 32%), and fatty (40% vs 12%) than the Big Mac®, and the Delicious Burger 2 as meaty (66% vs 42%) and smoky (47% vs. 4%) than the Big Mac® (*n* = 101; paired comparisons, *p* < 0.05) Together, these examples validate the generative AI model by demonstrating its ability to internalize canonical burger recipes and generate novel, delicious, and palatable designs.

### Generative AI creates sustainable burgers

**Fig. 3:**
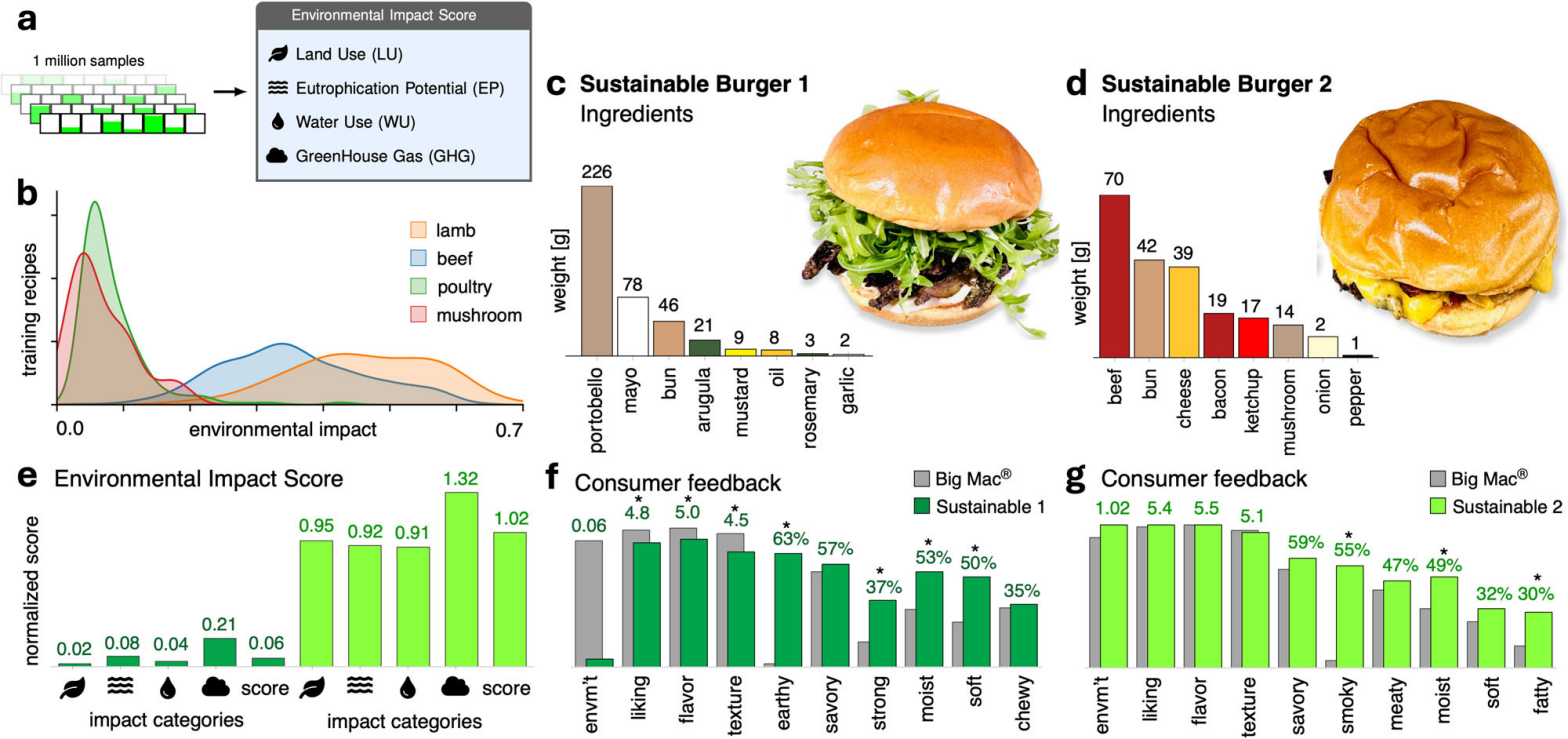
Generating Sustainable Burgers. The generative AI generates sustainable burgers from random AI generated samples, where the environmental impact score defines the collective impact of all ingredients in the recipe on land use, eutrophication potential, scarcity-weighted water use, and greenhouse gas emissions (**a**); distribution of environmental impact score for all training recipes with lamb, beef, poultry, and mushroom (**b**); generated recipes of Sustainable Burger 1 and Sustainable Burger 2 (**c**, **d**); environmental impact scores of Sustainable Burger 1 and Sustainable Burger 2 (**e**); consumer feedback for Sustainable Burger 1 and Sustainable Burger 2 compared to the Big Mac® (**f**, **g**); * indicates statistical significance (*p* < 0.05).

We next evaluate whether the generative AI model can identify and generate burger recipes with reduced environmental impact while maintaining consumer acceptance (Fig. 3). We quantify sustainability using an environmental impact score that aggregates ingredient-level contributions from land use, eutrophication potential, scarcity-weighted water use, and greenhouse gas emissions (Fig. 3a). Analysis of the training data shows that environmental impact scores vary substantially across recipes of different primary protein sources, with lamb- and beef-based recipes exhibiting systematically higher impacts than poultry- and mushroom-based recipes (Fig. 3b). Sampling one million recipes from the model enables identification of sustainable burger candidates, illustrated by two representative examples, Sustainable Burger 1 and Sustainable Burger 2, which differ in ingredient composition and dominant protein source (Fig. 3c, d). Sustainable Burger 1, a mushroom-based formulation, achieves an environmental impact score of 0.06, more than one order of magnitude lower the Big Mac® with 0.93, whereas Sustainable Burger 2, a mushroom-beef blend, with 1.02 ranks comparable to the Big Mac®(Fig. 3e). Consumer feedback indicates that Sustainable Burger 1 scores modestly below the Big Mac® in overall liking, flavor, and texture, whereas Sustainable Burger 2 performs on par with the Big Mac® in these categories. Sensory evaluation indicates that the two sustainable burgers achieve consumer ratings that differ in systematic ways from those of the classic Big Mac® (Fig. 3f, g): Sustainable Burger 1 received significantly lower ratings than the Big Mac® for overall liking (4.8 ± 1.8 vs. 5.3 ± 1.5), flavor (5.0 ± 1.9 vs. 5.4 ± 1.5), and texture (4.5 ± 1.9 vs. 5.2 ± 1.5), whereas ratings for Sustainable Burger 2 did not differ significantly from the Big Mac® across these attributes (*n* = 101; *p* < 0.05). Participants more frequently described the Sustainable Burger 1 as earthy (63% vs. 2%), strong (37% vs. 14%), moist (53% vs. 32%), and soft (50% vs. 25%) than the Big Mac®, and the Sustainable Burger 2 as smoky (55% vs. 4%), moist (49% vs. 32%), and fatty (30% vs. 12%) (*n* = 101; paired comparisons, *p* < 0.05 for all reported attributes). Together, these results validate that the generative AI model successfully navigates the trade-off between sustainability and palatability and discovers burgers with markedly reduced environmental impact without compromising taste.

### Generative AI creates nutritious and personalized burgers

**Fig. 4:**
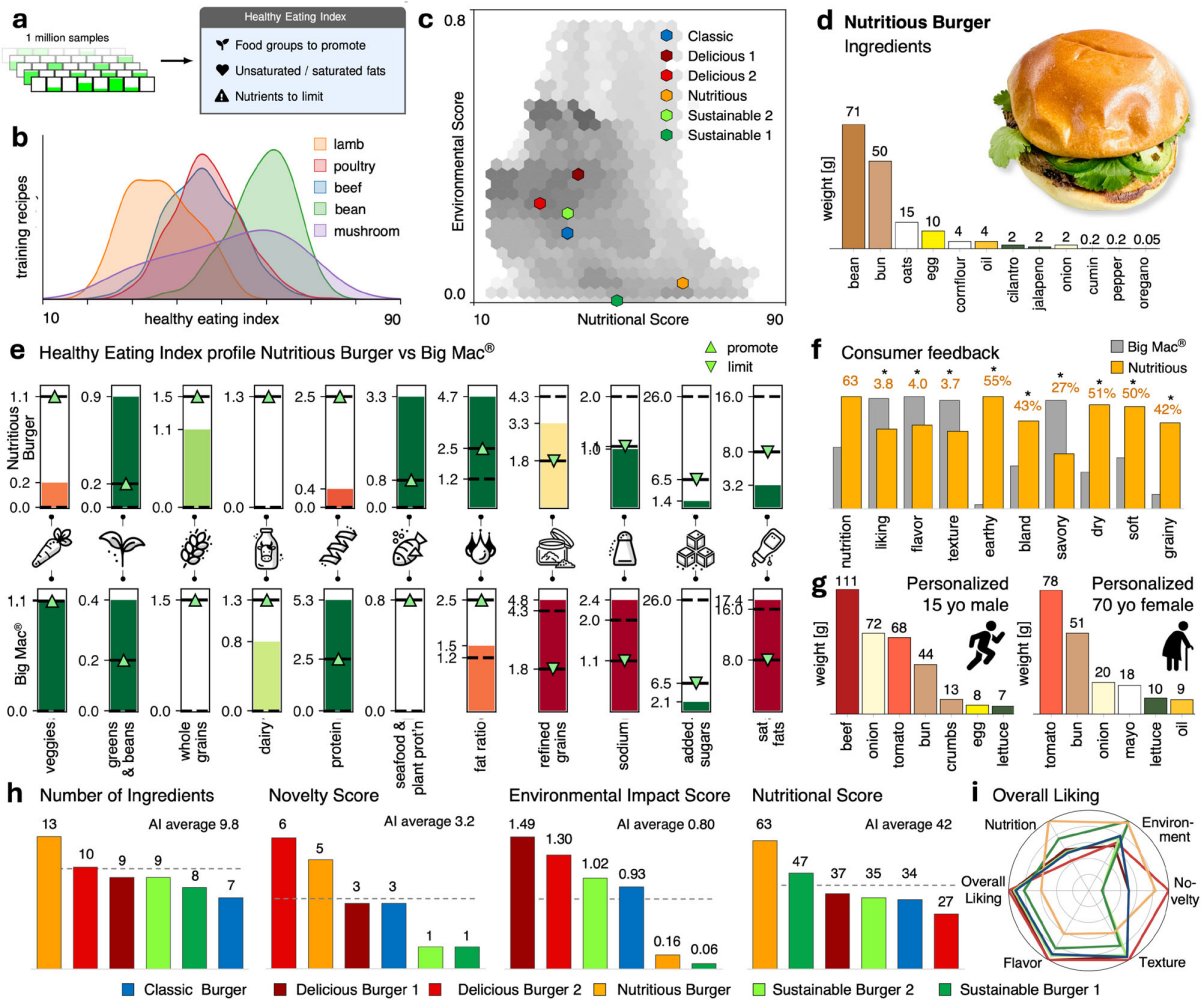
Generating Nutritious and Personalized Burgers. The generative AI generates nutritious burgers from random AI generated samples, where the healthy eating index defines the collective impact of all ingredients in the recipe on food groups to promote, unsaturated and saturated fats, and nutrients to limit (**a**); distribution of healthy eating index for all training recipes with lamb, poultry, beef, bean, and mushroom (**b**); nutritional-environmental score of all six burgers (**c**); generated recipe of Nutritious Burger (**d**); healthy eating index profile of Nutritious Burger compared to the Big Mac® (**e**); consumer feedback for Nutritious Burger compared to the Big Mac® (**f**); generated personalized recipes for 15-year-old, 180 cm, 80 kg active male and 70-year-old, 170 cm, 70 kg moderately active female (**g**); direct comparison of all six burgers by number of ingredients, novelty, environmental impact, and nutrition score (**h**); overall comparison (**i**); * indicates statistical significance (*p* < 0.05).

We next evaluate whether the generative AI model can identify burger recipes optimized for nutritional quality and adapt them to individual dietary needs (Fig. 4). We quantify nutrition using the healthy eating index, which aggregates contributions from food groups to promote, fatty acid composition, and nutrients to limit (Fig. 4a). Analysis of the training data reveals substantial variation in healthy eating index across recipes with different primary protein sources, with bean- and mushroom-based recipes exhibiting systematically higher nutritional scores than beef- and lamb-based recipes (Fig. 4b). Sampling one million recipes enables identification of a representative Nutritious Burger with a high nutritional score, which occupies a favorable region of the nutritional-environmental design space compared to the other AI generated burgers (Fig. 4c, d). The Nutritious Burger, a bean-based formulation, achieves a healthy eating index of 63.12, nearly twice as high as the Big Mac® with 33.71, while also reducing its environmental impact score by a factor of six (Fig. 4c). Relative to the Big Mac®, the Nutritious Burger shows improved alignment with dietary guidelines across multiple healthy eating index components, including increased contributions from vegetables, whole grains, and plant protein, alongside reduced refined grains, sodium, and saturated fat (Fig. 4e). Consumer feedback reveals a clear reduction in hedonic ratings for the Nutritious Burger relative to the Big Mac® (Fig. 4f): The Nutritious Burger received significantly lower ratings than the Big Mac® for overall liking (3.8 ± 1.7 vs. 5.3 ± 1.5), flavor (4.0 ± 1.8 vs. 5.4 ± 1.5), and texture (3.7 ± 1.8 vs. 5.2 ± 1.5) (*n* = 101; *p* < 0.05). Participants more frequently described the Nutritious Burger as earthy (55% vs. 2%), bland (43% vs. 21%), dry (51% vs. 18%), soft (50% vs. 25%), and grainy (42% vs. 7%), and less savory (27% vs. 53%) than the Big Mac® (*n* = 101; paired comparisons, *p* < 0.05 for all reported attributes). Beyond population-level optimization, the AI model also generates personalized burger recipes tailored to individual nutritional requirements. We demonstrate this feature by producing personalized recipes for a highly active 15-year-old male and a moderately active 70-year-old female, which differ in ingredient composition and quantities in accordance with age- and activity-specific dietary needs (Fig. 4g). Finally, a direct comparison across all six burgers highlights systematic trade-offs between ingredient count, novelty, environmental impact, and nutrition, and places the AI generated burgers in distinct regions of this multi-objective design space (Fig. 4h). In this design space, Delicious Burger 1 and Delicious Burger 2 score best in overall liking, flavor, and texture, whereas Sustainable Burger 1 and Nutritious Burger score best in nutrition and environment (Fig. 4i). Together, these results validate that the generative AI model can optimize for nutritional quality at both the population and individual levels while maintaining sensory acceptance.

## Discussion

### Learning the structure of food design

A central result of this study is that generative AI can learn the latent structure of food design directly from large-scale, human-generated recipe data^32^. Rather than reproducing superficial statistics, the model captures higher-order regularities that define burgers as a culinary class, including ingredient co-occurrence, typical recipe length, and characteristic quantity distributions. This perspective echoes broader findings from data-driven culinary science, such as the flavor network approach that uncovers principled patterns of ingredient co-occurrence across tens of thousands of recipes in global cuisines^33^. The ability to internalize the *statistical grammar of recipes* reflects the high-dimensional and nonlinear nature of human taste, which emerges from complex interactions among ingredients rather than simple additive rules^34^. Importantly, the rediscovery of classic benchmarks such as the Big Mac®–without explicit supervision–demonstrates that culturally dominant foods occupy high-probability regions of the learned design space and validates the model against widely recognized culinary reference points. This result is particularly remarkable given that the combinatorial recipe space spans 2^146^, i.e., more than 10^43^ possible ingredient combinations^22^. Together, these findings support the view that recipes encode collective human taste knowledge accumulated over decades, and that generative models can extract this knowledge to form an *interpretable and navigable design manifold*. This reframes food formulation from an artisanal, trial-and-error practice into a data-driven design science and positions recipes as a natural and human-centered interface between culinary tradition and artificial intelligence.

### Exploring novelty without sacrificing palatability

Beyond learning existing culinary structure, the generative model enables controlled exploration of novel burger designs while preserving sensory appeal. By explicitly quantifying novelty through a substantial difference score, we show that departures from canonical recipes do not inherently lead to diminished consumer acceptance. Instead, the model identifies regions of the design space where novelty and palatability coexist: It discovers burgers that differ meaningfully in ingredient composition and proportions while maintaining ratings for liking, flavor, and texture comparable to widely consumed references. This behavior reflects the *high-dimensional and nonlinear nature of human taste*, which emerges from complex interactions among ingredients and cannot be navigated reliably through intuition or simple empirical rules alone^20^. It also aligns with systematic analysis showing that consumer acceptance of novel foods depends on intrinsic sensory properties such as taste and familiarity as well as psychological factors such as neophobia and food experience^16^. The rediscovery of classic burgers alongside successful novel variants suggests that cultural familiarity anchors broad regions of acceptability within the culinary design space that enable innovation without alienating consumer expectations^35^. In this context, generative AI provides a *principled framework for exploring non-linear trade-offs* between familiarity and novelty, and reframes culinary innovation as navigation of a structured, data-driven design manifold rather than trial-and-error experimentation.

### Environmental sustainability as a system-level design objective

A key contribution of this work is demonstrating that generative AI can substantially reduce the environmental impact of burgers while remaining grounded in consumer-relevant designs. Livestock production is a major driver of greenhouse gas emissions, land use, and biodiversity loss^36^; yet, consumer adoption of lower-impact alternatives remains limited by taste, texture, and familiarity^37^. Our results show that sustainability emerges not from isolated ingredient substitutions, but from coordinated changes across entire recipes. This yields designs that achieve large reductions in environmental impact through *system-level rebalancing of ingredients*. This finding aligns with broader evidence that dietary sustainability depends on integrated food-system redesign rather than single-ingredient replacements^38^. Notably, the mushroom-only burger achieves an environmental impact score more than an order of magnitude lower than that of a conventional reference burger, while the mushroom-blended burger retains comparable impact and sensory performance. This highlights that environmental performance depends on interactions among ingredients, rather than any single component, and that generative models are well-suited to navigating such non-linear, multi-objective trade-offs. By embedding life-cycle considerations directly into the generative process, the model identifies sustainable designs that remain recognizable as burgers and addresses a central bottleneck in alternative protein adoption–*acceptance rather than availability*–a challenge repeatedly emphasized in studies of consumer response to plant-based and hybrid meat alternatives^39^. Together, these findings bridge the gap between environmental metrics and consumer-facing design, and position generative AI as a practical tool for system-level optimization of food sustainability.

### Nutrition and personalization as explicit design criteria

An additional contribution of this work is demonstrating that nutritional quality can be treated as an explicit, quantitative design objective, rather than a secondary outcome of food formulation. By optimizing generated recipes with respect to the healthy eating index^31^, the model identifies burgers that substantially outperform widely consumed benchmarks, achieving nearly twice the nutritional score of a conventional reference burger, while simultaneously reducing environmental impact by a factor of six. This result underscores that improvements in nutrition and sustainability need not be mutually exclusive, but instead emerge from coordinated, system-level adjustments across ingredient composition and quantities^38^. Importantly, the AI-generated nutritious burger achieves this performance using recognizable whole-food ingredients and a relatively short ingredient list, which directly addresses growing *concerns around ultra-processed foods* and additive-heavy formulations that undermine consumer trust. Extending beyond population-level optimization, the AI model demonstrates the ability to translate dietary reference intakes into concrete, consumer-facing food designs by *personalizing recipes based on age, sex, and activity level*, building on earlier initiatives to personalize nutrition based on glucose levels^40^. Together, these findings position generative AI as a practical bridge between nutritional science and everyday eating and enable objective comparison, optimization, and personalization of foods within a unified design framework.

### Limitations and scope

This study has several limitations that define the scope of the reported results. First, the generative model learns from existing, human-designed recipes and therefore inherits cultural, regional, and temporal biases present in the source data, which primarily reflect Western-style burger traditions. Second, the recipe representation includes only ingredient identities and quantities and does not explicitly account for processing steps, cooking methods, physicochemical transformations, or water redistribution during cooking that influence texture and flavor. As a result, the current framework cannot guarantee reproducibility of specific culinary profiles without standardized preparation protocols, culinary expertise, and additional physicochemical characterization. Third, the environmental and nutritional scores rely on aggregated databases and global averages and do not reflect variability associated with specific production practices, energy systems, agricultural methods, or supply chains. Accordingly, we should interpret the reported environmental impacts as comparative estimates rather than universal or absolute values. Fourth, sensory validation focused on a limited set of generated burgers and participants, and broader studies will be required to establish generalizability across populations and contexts. Despite these constraints, the model provides a flexible and extensible framework for multi-objective food design.

### Broader implications and future outlook

This work demonstrates how generative AI can shift food formulation from artisanal trial-and-error toward a quantitative, data-driven design science^32^. By learning directly from large-scale recipe data, the AI model functions as a domain-specific world model of contemporary burger design as it captures the statistical regularities and trade-offs that shape plausible and desirable foods. This enables systematic exploration of deliciousness, nutrition, and sustainability while remaining grounded in cultural familiarity, where adoption–rather than novelty–emerges as the central challenge in food innovation^39^. More generally, food offers a uniquely human-centered domain for generative AI, where models can align optimization objectives with sensory feedback, health, and environmental constraints^21^. Future work should extend this framework beyond ingredient selection and ingredient quantification toward richer representations that include processing, cooking, physicochemical properties, sensory descriptors, cost, supply-chain variability, and consumer subgroups. Such extensions would connect generative recipe design more directly to related efforts in data-driven culinary science^33^, alternative protein development^19^, sustainable diet modeling^38^, and personalized nutrition^40^. Ultimately, these advances could support end-to-end food design and establish generative AI as a collaborative tool at the intersection of human creativity, engineering, and planetary health.

## Discussion

This work shows that generative AI can reimagine food formulation as a quantitative, data-driven design process rooted in human preferences and measurable constraints. By learning directly from large-scale recipe data, the model captures the structure of burger design within the cultural and culinary scope of the training data and enables systematic exploration across taste, nutrition, and sustainability. Consumer feedback validates the generative model by confirming that its designs align with human taste preferences while delivering substantial improvements in nutritional quality and environmental impact relative to conventional benchmarks. Beyond burgers, this approach points toward a new paradigm for AI-driven food design that unites culinary creativity with human and planetary health.

## Methods

### Model architecture, training and validation

Food recipes are hybrid discrete–continuous objects that require *ingredient selection* and *ingredient quantification*. Here we represent each burger recipe solely by its ingredients and their associated weights. Accordingly, a recipe **x**_0_ = {**m**, **w**}, consists of a binary ingredient mask **m** ∈ {0, 1}^*K*^ that indicates presence or absence of each ingredient and the ingredient weight ${\bf{w}}\in {{\mathbb{R}}}^{K}$. We adopt a two-stage diffusion-based framework that decouples ingredient selection from ingredient quantification: Specifically, we integrate a *multinomial diffusion model*^41^ to generate ingredient masks with a *score-based generative model*^42^ to generate ingredient weights conditional on a given mask (Fig. 1a, Supplementary Material S.1.2).

#### Diffusion-based recipe generation

Conceptually, diffusion models learn burger design by progressively randomizing existing recipes into noisy ingredient combinations and then learning how to reverse this process to reconstruct realistic burgers^22^. Once trained, the model starts from random noise and iteratively samples plausible ingredient combinations and quantities that resemble human-designed recipes. Formally, diffusion models define a *forward stochastic process* *q*(**x**_*t*_∣**x**_*t*−1_) that gradually adds noise to data samples **x**_0_ to produce latent variables **x**_*t*_ that increasingly obscure the original data throughout *t* = 1, . . . , *T* time steps^43^. The learnable component is the *reverse stochastic process* *p*(**x**_*t*−1_∣**x**_*t*_), which progressively removes noise and enables generation from an unstructured prior. We train the diffusion model by maximizing a variational lower bound on the data likelihood, which corresponds to the evidence lower bound,

$$\log\,{\rm{P}}({{\bf{x}}}_{0}) \ge {{\mathbb{E}}}_{{{\bf{x}}}_{1},\cdots,{{\bf{x}}}_{T} \sim q}\left[\log p({{\bf{x}}}_{T})+\mathop{\sum }\limits_{t=1}^{T}\log \frac{p({{\bf{x}}}_{t-1}| {{\bf{x}}}_{t})}{q({{\bf{x}}}_{t}| {{\bf{x}}}_{t-1})}\right].$$

Here P(**x**_0_) denotes the marginal likelihood of an observed burger recipe under the generative model, obtained by integrating over all latent diffusion variables, where **x**_0_ is a human-designed burger recipe, **x**_*t*_ is its progressively noised representation, *q* is the fixed forward process, *p* is the learned reverse denoising process, and ${\mathbb{E}}$ is the expectation operator that denotes an average over noise realizations drawn from the forward diffusion process.

#### Ingredient selection via multinomial diffusion

We model ingredient selection using a multinomial diffusion process in which ingredient presence is treated as a categorical variable. We define the forward process as

$$q({{\bf{x}}}_{t}| {{\bf{x}}}_{t-1})={\mathcal{C}}\left({{\bf{x}}}_{t}| (1-{\beta }_{t}){{\bf{x}}}_{t-1}+{\beta }_{t}/K\right),$$

where ${\mathcal{C}}$ denotes a categorical distribution with the parameter listed after the vertical bar ∣, *β*_*t*_ controls the noise level at time step *t*, and *K* is the number of categories. In our application, ingredient selection is *binary*, *K* = 2, meaning an ingredient is either present or absent. The above equation reduces to a Bernoulli distribution with parameter (1 − *β*_*t*_)**x**_*t*−1_ + *β*_*t*_(1 − **x**_*t*−1_), which flips ingredient inclusion from present to absent or vice versa with a probability *β*_*t*_ and keeps it the same with a probability (1 − *β*_*t*_). As *t* increases, the ingredient mask becomes progressively randomized, while the learned reverse process reconstructs statistically plausible ingredient combinations, which inherently capture dependencies between ingredients that commonly co-occur in burger recipes.

#### Ingredient quantification via score-based diffusion

Conditional on a given ingredient mask, we generate ingredient quantities using a score-based generative model formulated through stochastic differential equations. The forward *noising process* is

$${\rm{d}}{{\bf{x}}}_{t}\,=\,f({{\bf{x}}}_{t},\,t)\,{{\rm{d}}t}\,+\,{g}(t)\,{{{\rm{d}}B}_{t}},$$

and the reverse-time *denoising process* is

$${\rm{d}}{{\bf{x}}}_{t}=\left[{g}^{2}(t){\nabla }_{{\bf{x}}}\log {p}_{t}({\bf{x}})-f({{\bf{x}}}_{t},t)\right]{\rm{d}}t+g(t)\,{\rm{d}}{\tilde{B}}_{t},$$

where *B*_*t*_ is a *K*-dimensional Brownian motion, ${\tilde{B}}_{t}$ is its time reversal, and *f* and *g* define the drift and diffusion coefficients^42,44^. Here, rather than learning the probability density *p*_*t*_(**x**) directly, we approximate the score function ${\nabla }_{{\bf{x}}}\log {p}_{t}({\bf{x}})$ using a neural network to enable efficient sampling of ingredient weights consistent with observed distributions in human-designed burger recipes.

#### Dataset and training

We train our model on a curated burger dataset derived from an open-source collection of over half a million human-designed recipes from Food.com^45,46^. We filter all recipes for burgers, extract ingredients, quantities, and units from free texts, and standardize and convert the data into a structured representation. The final dataset consists of 2,216 burger recipes made up of 146 ingredients (Suppl. Material S.1.1, Suppl. Tables 1 and 2).

#### Model validation and statistical fidelity

The trained model accurately reproduces both first-order and higher-order statistical properties of the training data: The *ingredient selection model* estimates the marginal probability of each ingredient appearing in a random burger recipe with a maximum absolute error below 1% (Fig. 1b). The *ingredient quantification model* predicts quantities in previously unseen recipes with a mean absolute error of 101.9 g (Fig. 1c), despite the extrapolatory and highly stochastic nature of the problem. Beyond marginal statistics, the model captures higher-order structure, including *pairwise ingredient correlations* (Fig. 1d) and *number of ingredients per recipe* (Fig. 1b, e). These properties are not explicitly enforced during training, but emerge from the learned generative process (Suppl. Material S.1.3, Suppl. Figs. 1 and 2).

#### Substantial difference score to quantify similarity

Quantifying the proximity between recipes and grouping similar recipes is useful for various applications, for example, to quantify the novelty of an AI-generated recipe. For this purpose, we define the semi-discrete *substantial difference score* between two recipes *r*_1_ and *r*_2_,

$${\mathrm{SDS}}({r}_{1},{r}_{2})=\mathop{\sum }\limits_{i=1}^{{n}_{{\rm{ing}}}}{d}_{i}({r}_{1},{r}_{2}),$$

as the sum of the binary distance *d*_*i*_ over all *n*_ing_ = 146 ingredients in the database, with

$${d}_{i}({r}_{1},{r}_{2})=\left\{\begin{array}{ll}1\quad\,{\mathrm{if}}\,\,{r}_{1i}+{r}_{2i}\ne 0\,\mathrm{and}\,{r}_{1i}\cdot {r}_{2i}=0\\ 1\quad\,{\mathrm{if}}\,\,\max ({r}_{1i},{r}_{2i})/\min ({r}_{1i},{r}_{2i})\ge 2\\ 0\quad\,{\mathrm{otherwise}}\,.\end{array}\right.$$

During *rediscovery*, we use the substantial difference score of zero, *SDS* = 0, to quantify a match between an AI-generated and a human-designed recipe. During *discovery*, we use values larger than zero, *SDS* > 0, to quantify the novelty of an AI-generated recipe compared to the human-designed recipes in the training set (Suppl. Material S.1.5).

#### Popularity score to quantify palatability

Palatability refers to qualities that make a food item desirable to the human palate, such as flavor, aroma, and texture. While the human palate displays significant variations across individuals, we can still quantify the overall palatability of a food product by measuring its *popularity score* within the population. This even extends to patterns in food preparation, as evidenced by the popularity of some combinations of ingredients compared to others (Fig. 1d). Here we propose to use popularity as a proxy for palatability. Our AI model learns the probability distribution of the human palate and assigns higher probabilities to popular recipes, patterns, and combinations. At the recipe level, more frequent repetitions effectively translate into a more palatable recipe associated with a higher popularity score.

### Generative AI for burgers

**Fig. 5:**
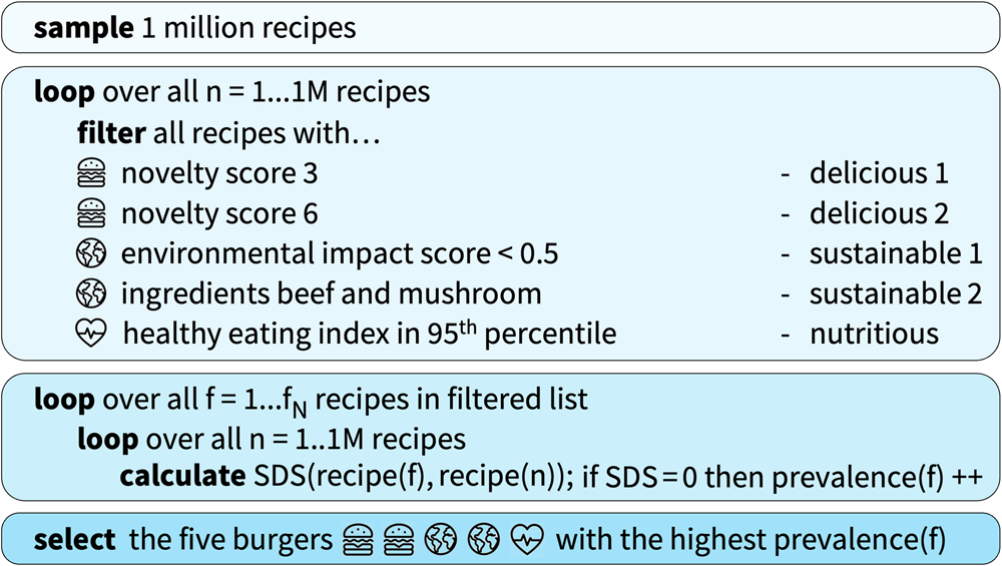
Generative AI for Burgers. Algorithmic workflow for selecting the five AI-generated burgers used in the sensory survey. We first sample one million burger recipes from the trained diffusion model and filter recipes for deliciousness, sustainability, and nutrition. For each filtered subset, we compute the substantial difference score (SDS) relative to all one million recipes and quantify recipe prevalence through the number of SDS = 0 matches. We then select the burgers with the highest prevalence within each design category under the hypothesis that the most prevalent recipes are the most palatable.

We use the trained and validated model to *rediscover* the classic Big Mac® and *discover* five new delicious, sustainable, and delicious burgers (Fig. 5).

#### The Classic burger

As a proof of concept, we use the generative model to *rediscover* the Big Mac®, one of the most widely consumed burgers worldwide^27^, served in more than 100 countries^28^. This global adoption reflects a high degree of palatability across diverse populations and makes the Big Mac® a stringent benchmark for evaluating whether the model captures widely shared preferences. Because the official recipe is proprietary, we approximate it by synthesizing four independent open-source recreations into a unified reference recipe^47–50^ (Fig. 1b, Suppl. Figs. 3 and 11). We then search for this reference recipe in randomly generated samples from the model (Fig. 1e). We define rediscovery as a sample with a substantial difference score of zero, SDS = 0, relative to the reference recipe. The training dataset did not contain the reference Big Mac® recipe (Supplementary Material S.1.4).

#### The delicious burger

Next, we use our artificial intelligence to *discover* delicious burgers, with a pre-defined *novelty score*. Specifically, we adopt the substantial difference score to quantify the novelty of an AI-generated sample by comparing it to the human-designed recipes in the training set (Suppl. Material S.1.5). For the Delicious Burger 1, we draw 1 million samples, filter all samples with *SDS* ≥ 3, and select the most repeated sample in the this list as the most palatable recipe with the highest popularity score (Fig. 2c, f, Supplementary Fig. 12). For the Delicious Burger 2, we perform the same steps, but now with *SDS* ≥ 6 (Fig. 2d, g, Suppl. Fig. 13).

#### The sustainable burger

We characterize environmental sustainability using life cycle assessment data, which estimate the total environmental impact of agricultural products across production and distribution chains based on global producer surveys^51^. We obtain ingredient-level data from a harmonized environmental database across *n* = 570 studies^36^. Since this database does not include mushrooms, we supplement it with land-use data from the United States Department of Agriculture^52^ and freshwater eutrophication potential, scarcity-weighted water use, and greenhouse gas emissions from European mushroom production^53^. We quantify sustainability using a single *environmental impact score* that averages normalized land use, aquatic eutrophication potential, scarcity-weighted water use, and greenhouse gas emissions across ingredients, weighted by their quantities^30^ (Suppl. Material S.1.6, Suppl. Table 3, Suppl. Figs. 4 and 5). For the Sustainable Burger 1, a plain mushroom burger with an environmental impact sore of 0.06, we draw 1 million samples, sort them by their environmental impact score, and select the most repeated recipe overall (Fig. 3c,f, Suppl. Fig. 14). For the Sustainable Burger 2, a beef-mushroom blend with an environmental impact sore of 1.02, we perform the same steps, but now select the most repeated recipe that contains both beef and mushroom (Fig. 3d, g, Suppl. Fig. 15).

#### The nutritious burger

We quantify nutritional quality using established nutritional profiling models that compare food and nutrient composition against dietary guidelines, including the healthy eating index^31^, nutri-score^54^, and health star rating^55^. Here we use the *healthy eating index* developed by the U.S. Department of Agriculture to assess alignment with the Dietary Guidelines for Americans and emphasizes food-group adequacy rather than individual nutrients^56,57^. We obtain food-group equivalents from the USDA Food Patterns Equivalents Database^58^ and nutrient composition data from USDA FoodData Central^59^, and compute the healthy eating index by aggregating ingredient-level food-group and nutrient contributions, normalized to 500 kcal servings (Suppl. Material S.1.7, Suppl. Figs. 6 and 7). For the Nutritious Burger, a bean-based formulation with a healthy eating index of 63.12, we draw 1 million samples, sort them by their healthy eating index (Fig. 4e), and select the most repeated recipe within the top 5% (Fig. 4d, f, Suppl. Fig. 16).

#### The personalized burger

We account for inter-individual variation in nutritional requirements using a personalized nutrient profiling model that tailors recipes to age, sex, body composition, and physical activity level^60^. We compute a *personalized nutrition score* on a 0-100 scale using individual characteristics, including age, sex, body weight, height, and physical activity level. We derive nutrient-specific target ranges from dietary reference intakes and acceptable macronutrient distribution ranges^61,62^, together with World Health Organization guidelines on upper intake limits for sodium, free sugars, and saturated fats^63–65^. We aggregate these targets into a single personalized nutrition score for each burger (Suppl. Material S.1.7). Using this framework, we generate personalized burger recipes for two representative demographic profiles, a 15-year-old, 180 cm, 80 kg active male and a 70-year-old, 170 cm, 70 kg moderately active female (Fig. 4g) and additional personalized recipes (Supplementary Fig. 8).

### Burger validation

#### Burger preparation

Our AI-generated recipes specify ingredients and quantities only, and do not include the processing or cooking steps needed to prepare the actual burgers. We therefore engage an executive chef to translate each ingredient list into standardized preparation, cooking, and assembly protocols, including ingredient handling, cutting, seasoning, cooking method, and burger assembly (Suppl. Material S.3). We then provide these finalized protocols to an independent group of chefs, who prepare the five AI-generated burgers and obtain the original Big Mac® for comparison in the sensory survey (Suppl. Material S.4).

#### Sensory survey

We conduct a blind sensory evaluation with *n* = 101 voluntary participants from the general population at an active restaurant in San Francisco, CA, in accordance with Stanford University Institutional Review Board guidelines (Supplementary Fig. 9). Each participant evaluates all six burgers on a 7-point Likert scale for *overall liking*, *flavor*, and *texture*^66^, and answers check-all-that-apply questions for *12 flavor-* and *15 texture*-related attributes (Suppl. Material S.2, Supplementary Data). All survey responses are fully anonymized.

#### Demographics

Of the *n* = 101 participants, 47.5% are male, 47.5% female, 3% non-binary, and 2% prefer not to say; 22% are 18–25 years old, 26% are 26–35, 19% are 36–45, 18% are 46–55, and 16% are older than 55; 65% are omnivores and 35% are flexitarians; the highest degree of education of 4% is a high school degree, 24% college, 50% batchelor’s, 11% master’s, 8% Ph.D. or higher, and 3% trade school; 4% eat burgers every day, 20% 2–3 times per week, 31% once a week, 27% 2–3 times per month, 16% every 1–2 months, and 3% 4–5 times per year (Supplementary Fig. 10).

#### Power and sample size

We select the number of participants, *n* = 101, to balance feasibility with statistical power. For the Likert-scale ratings, this sample size enables detection of small-to-moderate effect sizes using two-sided Welch’s t-tests. For the binary sensory attributes, the sample size provides > 80% power to detect differences of more than 20% between burgers at a significance level of *p* < 0.05 using paired comparisons. We did not perform an a priori power calculation; however, the sample size of *n* = 101 is comparable to or larger than those commonly used in consumer surveys of food products.

#### Statistical analysis

We report sensory ratings for overall liking, flavor, and texture on a 7-point Likert scale as mean ± standard deviation. We use two-sided Welch’s t-tests to compare the AI-generated burgers against the Big Mac®. We report binary flavor and texture attributes as percentage values and perform paired comparisons to assess statistical significance using two-sided binomial tests. We do not correct for multiple comparisons, as all tests were planned and hypothesis-driven. We report statistical significance as *p* < 0.05 (Figs. 2f, g, 3f, g, 4f).

## Supplementary information

Supplementary information

Supplementary Data

## Data Availability

The data of this study are available as Supplemental Material. The data of this study are also available at https://github.com/LivingMatterLab/AI4Food/tree/main/AI4Burgers/data.

## Code availability

The codes of this study are available at https://github.com/LivingMatterLab/AI4Food/tree/main/AI4Burgers. The generative model to create personalized burgers is available at https://ai4burgers.com.

## References

[1] Editorial. Diets, health and the environment. *Nat. Food* **5**, 717 (2024). 10.1038/s43016-024-01055-1 39313685

[2] Vermeulen, S. J., Campbell, B. M. & Ingram, J. S. Climate change and food systems. *Annu. Rev. Environ. Resour.* **37**, 195–222 (2012).

[3] Foley, J. A. et al. Global consequences of land use. *Science* **309**, 570–574 (2005). 16040698 10.1126/science.1111772

[4] Wada, Y. et al. Global depletion of groundwater resources. *Geophys. Res. Lett.* **37**, L20402 (2010).

[5] Xu, X. et al. Global greenhouse gas emissions from animal-based foods are twice those of plant-based foods. *Nat. Food* **2**, 724–732 (2021). 37117472 10.1038/s43016-021-00358-x

[6] Springmann, M. et al. Options for keeping the food system within environmental limits. *Nature* **562**, 519–525 (2018). 30305731 10.1038/s41586-018-0594-0

[7] Herforth, A. W., Bai, Y., Venkat, A. & Masters, W. A. The healthy diet basket is a valid global standard that highlights lack of access to healthy and sustainable diets. *Nat. Food* **6**, 622–631 (2025). 40425775 10.1038/s43016-025-01177-0 PMC12185308

[8] Canton, H. In Food and Agriculture Organization of the United Nations-FAO 23 edn 297–305 (Europa Publications, London, 2021).

[9] He, P. et al. Health-environment efficiency of diets shows nonlinear trends over 1990–2011. *Nat. Food* **5**, 116–124 (2024). 38332359 10.1038/s43016-024-00924-z PMC10896724

[10] Popkin, B. M. The nutrition transition in low-income countries: an emerging crisis. *Nutr. Rev.* **52**, 285–298 (2009). 10.1111/j.1753-4887.1994.tb01460.x 7984344

[11] Aune, D., Ursin, G. & Veierød, M. B. Meat consumption and the risk of type 2 diabetes: a systematic review and meta-analysis of cohort studies. *Diabetologia* **52**, 2277–2287 (2009). 19662376 10.1007/s00125-009-1481-x

[12] Lopez Barrera, E. & Hertel, T. Solutions to the double burden of malnutrition also generate health and environmental benefits. *Nat. Food* **4**, 616–624 (2023). 37488342 10.1038/s43016-023-00798-7

[13] Huang, T. et al. Cardiovascular disease mortality and cancer incidence in vegetarians: a meta-analysis and systematic review. *Ann. Nutr. Metab.* **60**, 233–240 (2012). 22677895 10.1159/000337301

[14] Lappé, F. M. Diet for a Small Planet. Ballantine Books: New York, (1971).

[15] Friedrich, B. Transforming a 12,000-year-old technology. *Nat. Food* **3**, 807–808 (2022). 37117888 10.1038/s43016-022-00604-w

[16] Laureati, M., Delgado, A. M., De Boni, A. & Sinesio, F. Determinants of consumers’ acceptance and adoption of novel food in view of more resilient and sustainable food systems in the EU: a systematic literature review. *Foods* **13**, 1534 (2024). 38790835 10.3390/foods13101534 PMC11120339

[17] St. Pierre, S. R. & Kuhl, E. Mimicking mechanics: a comparison of meat and meat analogs. *Foods* **13**, 3495 (2024). 39517278 10.3390/foods13213495 PMC11545010

[18] Mellor, C. et al. Consumer knowledge and acceptance of “algae” as a protein alternative: a UK-based qualitative study. *Foods* **11**, 1703 (2022). 35741901 10.3390/foods11121703 PMC9223121

[19] van den Bedem, S. D., Cotto, C. & Kuhl, E. Open-source benchmarking of plant-based and animal meats. *Foods***15**, 2112 (2026).42354080 10.3390/foods15122112

[20] Spence, C. Multisensory flavor perception. *Cell* **161**, 24–35 (2015). 25815982 10.1016/j.cell.2015.03.007

[21] Kuhl, E. AI for food: Accelerating and democratizing discovery and innovation. science of food. *npj Sci. Food* **9**, 82 (2025). 40404647 10.1038/s41538-025-00441-8 PMC12098880

[22] Tac, V. & Kuhl, E. Generative AI for material design: A mechanics perspective from burgers to matter. *Comp. Meth. Appl. Mech. Eng.* **461**, 11971 (2026).

[23] OpenAI. GPT-4 Technical Report. *arXiv*10.48550/arXiv.2303.08774 (2023).

[24] Vaswani, A. et al. Attention is all you need. *Adv. Neural Info. Proc. Syst.* **30**, 5998–6008 (2017).

[25] Thomas, A. T. et al. What can large language models do for sustainable food? 42nd International Conference on Machine Learning, PMLR 267: 59377-59433 (2025).

[26] Brown, T. B. et al. Language models are few-shot learners. *Adv. Neural Inf. Process. Syst.* **33**, 1877–1901 (2020).

[27] Spencer, E. H., Frank, E. & McIntosh, N. F. Potential effects of the next 100 billion hamburgers sold by McDonald’s. *Am. J. Preventive Med.* **28**, 379–381 (2005). 10.1016/j.amepre.2005.01.009 15831345

[28] McDonald’s delivers strong performance worldwide; November comparable sales rise 7.4%. PR Newswire (2011).

[29] Pakko, M. R. & Pollard, P. S. Burgernomics: a Big Mac™ guide to purchasing power parity. *Fed. Reserve Bank St. Louis Rev.* **85**, 9–28 (2003).

[30] Clark, M. et al. Estimating the environmental impacts of 57,000 food products. *Proc. Natl. Acad. Sci.* **119**, e2120584119 (2022). 35939701 10.1073/pnas.2120584119 PMC9388151

[31] Krebs-Smith, S. M. et al. Update of the Healthy Eating Index: HEI-2015. *J. Acad. Nutr. Dietetics* **118**, 1591–1602 (2018). 10.1016/j.jand.2018.05.021 PMC6719291 30146071

[32] Datta, B. et al. Artificial intelligence for food innovation. *Nat. Food*. 10.1038/s43016-026-01380-7 (2026). 10.1038/s43016-026-01380-7 42399700

[33] Ahn, Y.-Y., Ahnert, S. E., Bagrow, J. & Barabasi, A.-L. Flavor network and the principles of food pairing. *Sci. Rep.* **1**, 196 (2011). 22355711 10.1038/srep00196 PMC3240947

[34] Barabasi, A.-L., Menichetti, G. & Loscalzo, J. The unmapped chemical complexity of our diet. *Nat. Food* **1**, 33–37 (2020).

[35] Torrico, D. D., Fuentes, S., Gonzalez Viejo, C., Ashman, H. & Dunshea, F. R. Cross-cultural effects of food product familiarity on sensory acceptability and non-invasive physiological responses of consumers. *Food Res. Int.* **115**, 439–450 (2019). 30599962 10.1016/j.foodres.2018.10.054

[36] Poore, J. & Nemecek, T. Reducing food’s environmental impacts through producers and consumers. *Science* **360**, 987–992 (2018). 29853680 10.1126/science.aaq0216

[37] Godfray, H. D. J. et al. Meat consumption, health, and the environment. *Science* **361**, eaam5323 (2018). 10.1126/science.aam5324 30026199

[38] Willett, W. et al. Food in the anthropocene: the eat-lancet commission on healthy diets from sustainable food systems. *Lancet* **393**, 447–492 (2019). 30660336 10.1016/S0140-6736(18)31788-4

[39] Bryant, C. & Barnett, J. Consumer acceptance of cultured meat: A systematic review. *Meat Sci.* **143**, 8–17 (2020). 10.1016/j.meatsci.2018.04.008 29684844

[40] Zeevi, D. et al. Personalized nutrition by prediction of glycemic responses. *Cell* **163**, 1079–1094 (2015). 26590418 10.1016/j.cell.2015.11.001

[41] Hoogeboom, E., Nielsen, D., Jaini, P., Forré, P. & Welling, M.Ranzato, M., Beygelzimer, A., Dauphin, Y., Liang, P. & Vaughan, J. W. (eds) Argmax flows and multinomial diffusion: Learning categorical distributions. (eds Ranzato, M., Beygelzimer, A., Dauphin, Y., Liang, P. & Vaughan, J. W.) Advances in Neural Information Processing Systems, Vol. 34, 12454–12465 (2021).

[42] Song, Y. et al. Score-based generative modeling through stochastic differential equations. International Conference on Learning Representations (2021).

[43] Ho, J., Jain, A. & Abbeel, P.Larochelle, H., Ranzato, M., Hadsell, R., Balcan, M. & Lin, H. (eds) Denoising diffusion probabilistic models. (eds Larochelle, H., Ranzato, M., Hadsell, R., Balcan, M. & Lin, H.) Advances in Neural Information Processing Systems, Vol. 33, 6840–6851 (2020).

[44] Taç, V., Rausch, M. K., Bilionis, I., Sahli Costabal, F. & Tepole, A. B. Generative hyperelasticity with physics-informed probabilistic diffusion fields. *Eng. Comput.* **41**, 51–69 (2024). 40330696 10.1007/s00366-024-01984-2 PMC12052335

[45] Alvin. Food.com - Recipes and Reviews. https://www.food.com (2020).

[46] Wei, A. Food.com Recipes and Interactions. Kaggle dataset, https://www.kaggle.com/datasets/shuyangli94/food-com-recipes-and-user-interactions (2023).

[47] Stinson, T. The best homemade Big Mac recipe. online recipe, https://thegirlonbloor.com/homemade-big-mac-recipe (2024).

[48] Kaloudis, T. Big Mac. online recipe, https://www.theodorakaloudis.com/recipe-development (2024).

[49] Smith, K. Big Mac recipe. online recipe, https://www.bakingbeauty.net/copycat-big-mac (2024).

[50] Dimpflmaier, R. Make your own Big Mac. online recipe, https://www.fogocharcoal.com/blogs/cook/make-your-own-big-mac (2024).

[51] Hellweg, S. & Milà I Canals, L. Emerging approaches, challenges and opportunities in life cycle assessment. *Science* **344**, 1109–1113 (2014). 24904154 10.1126/science.1248361

[52] Mushrooms. Tech. Rep. ISSN: 1949–1530, National Agricultural Statistics Service (NASS) (2016).

[53] Goglio, P. et al. An environmental assessment of Agaricus bisporus ((J.E.Lange) Imbach) mushroom production systems across Europe. *Eur. J. Agron.* **155**, 127108 (2024).

[54] Julia, C., Etilé, F. & Hercberg, S. Front-of-pack Nutri-Score labelling in France: An evidence-based policy. *Lancet Public Health* **3**, e164 (2018). 29483002 10.1016/S2468-2667(18)30009-4

[55] Barrett, E. M. et al. Modifying the Health Star Rating nutrient profiling algorithm to account for ultra-processing. *Nutr. Dietetics* **82**, 53–63 (2025). 10.1111/1747-0080.12892 PMC11795220 38984976

[56] 2015-2020 Dietary Guidelines for Americans. Tech. Rep. 8th ed., US Department of Health and Human Services (USDHHS) and US Department of Agriculture (USDA) (2015).

[57] Herforth, A. et al. A global review of food-based dietary guidelines. *Adv. Nutr.* **10**, 590–605 (2019). 31041447 10.1093/advances/nmy130 PMC6628851

[58] Bowman, S. A., Clemens, J. C., Friday, J. E. & Moshfegh, A. J.Food Patterns Equivalents Database 2017-2018 Methodology and User Guide (U.S. Department of Agriculture, Beltsville, Maryland, 2020).

[59] U.S. Department of Agriculture (USDA). FoodData Central: Foundation Foods (Agricultural Research Service, 2024).

[60] Mainardi, F., Drewnowski, A. & Green, H. Personalized nutrient profiling of food patterns: Nestlé’s nutrition algorithm applied to dietary intakes from NHANES. *Nutrients* **11**, 379 (2019). 30759867 10.3390/nu11020379 PMC6412928

[61] Committee to Review the Dietary Reference Intakes for Sodium and Potassium, Food and Nutrition Board, Health and Medicine Division & National Academies of Sciences, Engineering, and Medicine. Dietary Reference Intakes for Sodium and Potassium (National Academies Press, Washington, D.C., 2019). 30844154

[62] Standing Committee for the Review of the Dietary Reference Intake Framework, Food and Nutrition Board, Health and Medicine Division & National Academies of Sciences, Engineering, and Medicine. Rethinking the Acceptable Macronutrient Distribution Range for the 21st Century: A Letter Report (National Academies Press, Washington, D.C., 2024). 39680697

[63] World Health Organization. *WHO Global Report on Sodium Intake Reduction* 1st edn (World Health Organization, Geneva, 2023).

[64] World Health Organization. Guideline: Sugars Intake for Adults and Children (World Health Organization, Geneva, 2015). 25905159

[65] World Health Organization. Saturated Fatty Acid and Trans-Fatty Acid Intake for Adults and Children: WHO Guideline (World Health Organization, Geneva, 2023). 37490572

[66] St Pierre, S. et al. The mechanical and sensory signature of plant-based and animal meat. *npj Sci. Food* **8**, 94 (2024). 39548076 10.1038/s41538-024-00330-6 PMC11568319

