# Supplementary material for: Generative artificial intelligence creates delicious, sustainable, and nutritious burgers
Source: NPJ Sci Food. 2026 Jun 26;10:199. doi: 10.1038/s41538-026-00953-x (PMC13309617; doi:10.1038/s41538-026-00953-x)
Supplement: Supplementary file 1 — Supplementary information [file 41538_2026_953_MOESM1_ESM.pdf]

Generative Artificial Intelligence creates delicious, sustainable, and nutritious burgers

# – Supplementary Material –

Vahidullah Tac[1*], Christopher Gardner[2] and Ellen Kuhl[1]

[1]Department of Mechanical Engineering, Stanford University, Stanford, USA.
[2]Prevention Research Center, Stanford University School of Medicine, Stanford, USA.

*Corresponding author(s). E-mail(s): vtac@stanford.edu;
Contributing authors: cgardner@stanford.edu; ekuhl@stanford.edu;

S.1. The Generative AI model

S.1.1. Data preparation

The dataset used in this study is distilled from open-source databases of recipes from `food.com` [1, 2]. The original database at [1] lacks the units of measurements of the ingredients, so we combine it with a complementary database [2]. The combined database consists of 522,517 recipes by 57,178 authors on various categories of foods. Supplementary Table 1 illustrates a typical recipe from the combined database.

Identifying burger recipes

There are a few approaches for identifying burger recipes, all with their own caveats. One approach is to simply select recipes that mention the phrase `burger` in the name. However, this fails for some entries such as `hamburger soup`, which is a soup but still contains the term `burger`, or `beef sliders`, which is a burger but does not contain the term `burger`. Another approach is to use a large language model. We use a combination of the two methods to maximize training data points and reduce false positives. For each one of the recipes, a large language model, Mistral 7b [3], is prompted to decide if a recipe is a burger recipe given its name, for each one of the recipes. A recipe is deemed a *burger recipe* if the result of the large language model search is positive positive *and* the name contains the term `burger`.

Parsing burger ingredients

As shown in Supplementary Table 1, the ingredients and their quantities and units of measurement are given in plain text. Some ingredient descriptions are non-standard, for example: quantities with multiples, e.g., two 0.5-quart cans; countable items without units, e.g., two eggs; missing quantities, e.g., some salt; ranges, rather than exact values, e.g., one to two tomatoes; and ingredients with descriptors, e.g., fresh oregano. This makes parsing the recipes challenging. To standardize heterogeneous ingredient descriptions across online recipes, we use a large language model to parse raw ingredient text into structured triplets,

$$\{\texttt{ingredient name}, \texttt{quantity}, \texttt{unit}\}\,.$$

Supplementary Table 1 Representative burger recipe. A typical recipe from the combined `food.com` database contains a recipe ID and name; author ID and name; cooking, preparation, and total times; publication date; description; images; recipe category; keywords; ingredients; recipe instructions; and scores. From a total of 522,517 recipes in the `food.com` database, we identify 3,882 burger recipes from which we generate our training data.

Recipe ID	2734
Name	Ken's Hamburgers
Author ID	1575
Author Name	lindaWWJD
Cook Time	10 min
Prep Time	10 min
Total Time	20 min
Date Published	1999-08-25 05:21:00
Description	Make and share this Ken's Hamburgers recipe from Food.com.
Images	https://img.sndimg.com/food/image/upload/w_555,h_416,c_fit, fl_progressive,q_95/v1/img/recipes/27/34/picgsfaLB.jpg, https://img.sndimg.com/food/image/upload/w_555,h_416,c_fit,fl_progressive,q_95/v1/img/recipes/27/34/picaPkIS9.jpg
Recipe Category	Meat
Keywords	Very Low Carbs, High Protein, Kid Friendly, High In..., Broil/Grill, ¡ 30 Mins, Oven, Easy
Ingredients (raw)	• 1/3 lb lean ground beef (per burger)<br>• 1 1/2 teaspoons barbecue sauce<br>• 1/4 teaspoon dehydrated onion, minced
Recipe Instructions	• Allow 1/3 lb. lean ground beef per burger.<br>• To each burger, add 1 1/2 teaspoons barbeque sauce and 1/4 teaspoon dehydrated minced onion.<br>• Cook on broiler, turning about every 4 minutes.<br>• Also great on the grill.<br>• Serve with more barbeque sauce, not catsup.
Aggregated Rating	4.0
Review Count	5
Calories	273.2
Fat Content	15.2
⋮	⋮

The model normalizes quantities to single numeric values, converts measurements to mass or volume when possible, averages reported ranges, and removes qualitative descriptors such as size or preparation. We then manually review and correct the parsed outputs to resolve any remaining inconsistencies. The resulting curated dataset contains 3,882 recipes with 3,683 unique ingredients expressed in 171 distinct units of measurement.

Unifying units of measurement

The units of measurement are processed further for finding commonalities and standardizing the data. Some of the processing steps include: unifying spellings of units of measurement, e.g., g vs gr or teaspoon vs tsp; eliminating recipes that use a unique unit of measurement from consideration; eliminating recipes that use non-standard units of measurement, e.g., can or package; and resolving formatting issues from parsing step. This results in a further simplification to 3,725 recipes utilizing 3,535 unique ingredients measured in 30 unique units of measurement.

Unifying ingredient names

At this stage, all ingredient names are manually processed to resolve large language model artifacts. Some examples include: simplifying texts, e.g., X becomes Y or X $\rightarrow$ Y instead of just Y; removing additional explanations from the large language model; removing special characters, e.g.,*, ", . converting plurals to

singulars, e.g., leaves to leaf or tomatoes to tomato; unifying multiple spellings of the same ingredient, e.g., chile and chili. The ingredient names are further streamlined by removing unnecessary descriptors: removing marketing terms and brand names, e.g., from Heinz ketchup to ketchup; grouping all proprietary seasoning mixes and rare seasoning mixes under the overarching term seasoning; combining all beef products, e.g., ground beef, steak, beef chuck, etc. are replaced by beef; combining chicken and pork products; combining variations of the same ingredient name, e.g., adobo and adobo sauce or artichoke and artichoke hearts; combining similar products into one, e.g., baking powder and baking soda; classifying all types of bread into one of three categories, bread, roll, and bun; and correcting misspellings. This resulted in a reduction to only 709 unique ingredients. Next, the recipe list is processed through a first frequency filter by removing all recipes that contain rare ingredients, used less than 10 times, since such low utilization is a barrier for learning about those ingredients. Then, the list of unique ingredients is further standardized including: combining vegetables and fruits into their overarching categories, e.g., kidney beans and pinto beans into beans; combining all vinegar types; combining all liquid oils; combining all fish; combining all types of bread and bun into bun; and eliminated water from the ingredients. Finally, the recipe list is processed through a second frequency filter by removing all recipes that contain rare ingredients, used less than 15 times. At the end of this step, the final curated dataset consists of 2218 recipes and 146 unique ingredients.

Converting units of measurement into grams

The objective of this step is to replace all units of measurement with grams. For this, the units of measurement undergo the following processing steps: if the quantity is not available, the unit of measurement is removed; all units of volume, e.g., gallons, liter, tablespoon, teaspoon, are converted to milliliter; all units of weight, e.g., kilogram, pound, ounce, are converted into gram; and misattributions of the large language model are manually corrected, e.g., from one cube of beef to one cube of beef bouillon. Next, all ingredient volumes are converted into ingredient weights, from units of milliliter to gram, using density values from food density databases [4–6]. Finally, all remaining measurement units are converted into grams [7]. To do so, ingredient names are paired with unique USDA FDC IDs by searching the database for the ingredient name, and selecting the closest match. This step was facilitated by the `usda_fdc` package in python. The search results were sorted with the following priorities: exact matches; matches with least descriptors, e.g., bouillon cube is preferred over beef bouillon cube when searching for bouillon; matches from `Foundation foods` category; and matches from `SR Legacy foods` category. Supplementary Table 2 summarizes the final list of ingredients and their FDC IDs.

Processing ingredient quantities

The automated processing steps with code or large language models detailed so far can result in erroneous entries such as non-numeric entries for quantities, e.g., 1/2 instead of 0.5. These are corrected through manual processing. The recipes are then normalized by calories such that the ingredients of each recipe sum up to 500 kcal using the calorie density of each ingredient from the FDC database. At this final stage, some ingredients in the database can still have missing quantities. We replace these missing values with the average quantity across all recipes that utilize that ingredient. All these steps collectively result in the final `burgers database` where each recipe is represented through

$$\{\texttt{ingredient name}; \texttt{weight in grams}\}\,.$$

and stored in a 2,216 x 146 array, where the columns correspond to recipes, and the rows correspond to the quantities of the ingredients. Supplementary Table 2 summarizes all 146 ingredients and their corresponding USDA FDC codes.

Supplementary Fig. 1 highlights some statistics of the data including the top 10 most used ingredients by frequency and weight. Supplementary Fig. 2 displays typical relative distributions of weight for the top 10 most used ingredients by frequency in Supplementary Fig. 1a.

S.1.2. Modeling

In the current study, a recipe refers to the selection of ingredients and their quantities to be used in a burger. There are a total of 146 unique ingredients (Supplementary Table 2), and each recipe is a combination of a few of the ingredients in this list. A typical recipe has around ten ingredients and rarely exceeds twenty

Supplementary Table 2 List of all 146 ingredients used in this study. Burger ingredients are down-selected from an initial list of 3,683 by combining ingredient categories to a list of 709, filtered through a first frequency filter, again down-selected by combining ingredient categories, and filtered through a second frequency filter, resulting in the 146 ingredients listed in alphabetic order with their USDA FDC codes [7].

Ingredient name	FDC ID	Ingredient name	FDC ID	Ingredient name	FDC ID
allspice	171315	curry powder	170924	pasta	168927
almond	2707486	dill	172233	peanut	2515376
apple	2709215	dough	172791	pepper	170931
arugula	2709791	dressing	173592	pesto	171582
avocado	2709223	egg	171287	pickle	169379
bacon	168277	feta	173420	pimento	168559
barbecue sauce	174523	fish	2706284	pineapple	169124
basil	172232	flour	169761	pork	2705863
bean	173744	garlic	169230	portobello	169255
beef	2514743	ginger	169231	potato	170027
beer	168749	goat cheese	2705716	provolone	170850
beet	2685576	gouda	171241	quinoa	168874
blue cheese	172175	green onion	170005	ranch sauce	173592
bouillon	171562	greens	2709792	relish	168561
breadcrumb	1930877	guacamole	2709307	rice	168879
broth	172883	hoisin sauce	172886	rosemary	173473
brown sugar	2710260	honey	169640	sage	170935
buffalo	175299	horseradish	173472	salsa	174524
bulgur	2710820	hot sauce	171186	salt	173468
bun	2707784	italian seasoning	1887966	sauce	174524
butter	173430	jalapeno	168576	sausage	174584
cabbage	169975	ketchup	168556	sesame seed	170150
cajun seasoning	1887349	lamb	2705907	shallot	170499
carrot	2709660	lemon	2709168	sour cream	2346387
cayenne pepper	170932	lentil	172420	soy sauce	174278
celery	2709778	lettuce	2709789	spinach	2709614
cheddar cheese	2705709	lime	168155	steak sauce	171825
other cheese	2705764	liquid smoke	2680455	steak seasoning	2157193
chicken	2706091	mango	2709242	sugar	169655
chicken seasoning	2060346	marjoram	170928	sunflower seed	170562
chickpea	173756	mayonnaise	168112	taco seasoning	172243
chili	170497	milk	2705385	tahini	2707587
chili flake	579086	mint	173474	tarragon	170937
chip	2709434	monterey jack	2705720	teriyaki sauce	171167
chipotle chili	2009291	mozzarella	170845	thyme	173470
chive	2709781	mushroom	169251	tofu	172476
cilantro	169997	mustard	171043	tomato	170457
cinnamon	171320	noodle	2708354	tortilla	175036
coriander seed	170922	nutmeg	171326	turkey	171505
corn	2709783	oat	2708489	vinegar	172241
cornflour	2710835	oil	748278	walnut	170187
cottage cheese	172182	olive	2710089	wasabi	168583
cracker	2708184	onion	170000	wheat germ	168892
cranberry	2709279	orange	169918	wine	173185
cream	2705597	oregano	171328	worcestershire sauce	171610
cream cheese	173418	other seasoning	171331	yeast	167717
cucumber	2709784	paprika	171329	yogurt	171284
cumin	170923	parmesan	171247	zucchini	168565
curry	2710178	parsley	170416		

(Fig. 1, main manuscript). Modeling such data is challenging, as it represents a combination of *discrete* and *continuous* data. Here we propose a sequential two step approach: first, identify the ingredients that will be used in a recipe using a *mask model*; second, determine the quantities of the chosen ingredients using a *value model*. The first step is a discrete modeling process, while the second step deals with continuous data. We use a multinomial diffusion modeling for the ingredient mask, and a score-based generative model for the ingredient weights.

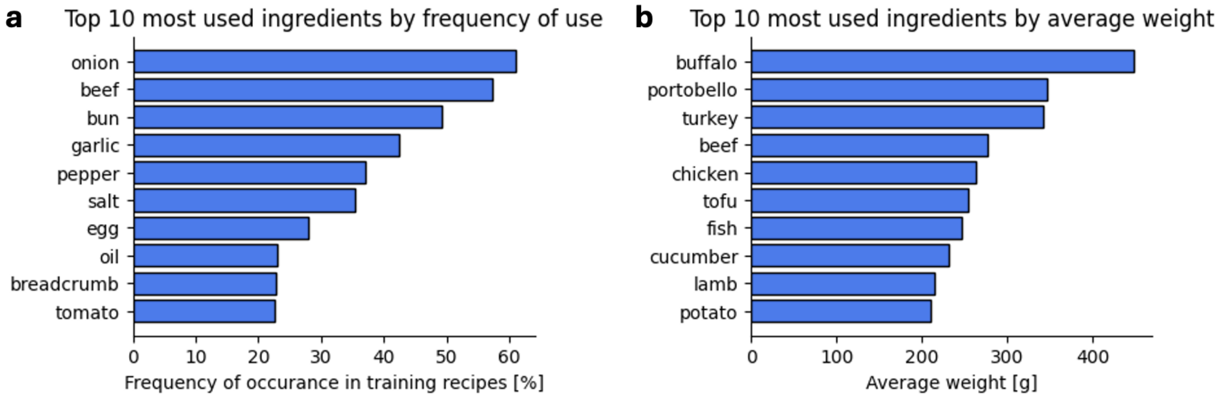

Supplementary Figure 1 **Top 10 most used ingredients**. Most used ingredients, out of a total of 146 ingredients, in the 2,216 recipes of our training data, sorted by frequency of use, with onion, beef, and bun being part of more than half of all recipes (a) and by average weight, with buffalo, portobello, and turkey contributing more than 300 g (b).

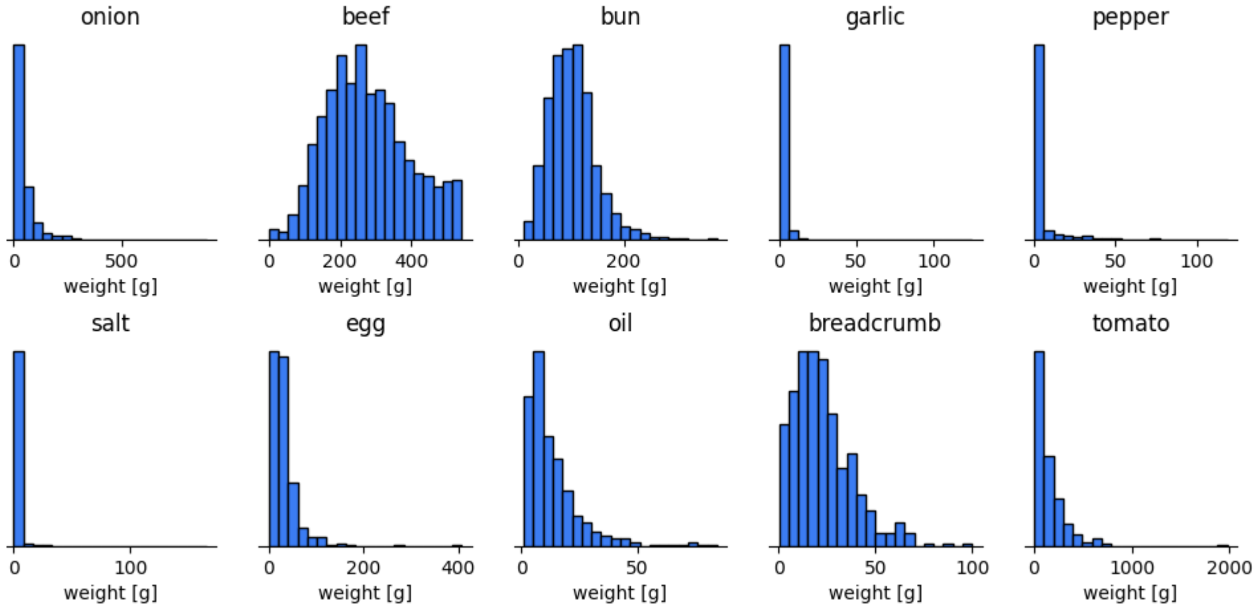

Supplementary Figure 2 **Relative distribution of weight of the top 10 ingredients.** Most used ingredients, out of a total of 146 ingredients, in the 2,216 recipes of our training data, sorted by frequency of use (Supplementary Fig. 1a), reported by their relative distributions.

Mask model

In multinomial diffusion [8], the diffusion process is defined in terms of categorical distributions that has a β_t chance of resampling a category uniformly,

$$q(\mathbf{m}_t|\mathbf{m}_{t-1}) = \mathcal{C}(\mathbf{m}_t|(1-\beta_t)\mathbf{m}_{t-1} + \beta_t/K)\,. \tag{1}$$

As a result, when using a small β_t, this has a large probability of resampling the same category as $\mathbf{m}_{t-1}$ and a small probability of uniformly selecting a differing category. Since these distributions form a Markov chain, the distribution at any t can be given in terms of the initial data $\mathbf{m}_0$ as

$$q(\mathbf{m}_t|\mathbf{m}_0) = \mathcal{C}(\mathbf{m}_t|\bar{\alpha}_t\mathbf{m}_0 + (1-\bar{\alpha}_t)/K)\,, \tag{2}$$

where $\bar{\alpha}_t = \prod_{\tau=1}^{t}(1-\beta_\tau)$. The posterior distribution has the following form,

$$q(\mathbf{m}_{t-1}|\mathbf{m}_t, \mathbf{m}_0) = \mathcal{C}(\mathbf{x}_{t-1}|\boldsymbol{\theta}_{\text{post}}(\mathbf{m}_t, \mathbf{m}_0)) \quad \text{with} \quad \boldsymbol{\theta}_{\text{post}} = \boldsymbol{\theta}/\sum_{k=1}^{K}\theta\,, \tag{3}$$

where $\boldsymbol{\theta}_{\text{post}}$ is the normalized vector of the weight vector $\boldsymbol{\theta}$ that combines information from the noisy state $\mathbf{m}_t$ and the origin data $\mathbf{m}_0$,

$$\boldsymbol{\theta} = [(1-\beta_t)\mathbf{m}_t + \beta_t/K] \odot [\bar{\alpha}_{t-1}\mathbf{m}_0 + (1-\bar{\alpha}_{t-1})K]\,, \tag{4}$$

where $\odot$ signifies element-wise Hadamard product. Contrary to conventional diffusion models that predict the parameters for predicting $\mathbf{m}_{t-1}$ directly, we instead model the noise which provides an explicit form for the posterior mean and results in a well conditioned loss [9]. Using this approach, we estimate $\mathbf{m}_0$ from $\mathbf{m}_t$ using a neural network $\hat{\mathbf{m}}_0 = \mu(\mathbf{m}_t, t)$. Then, the posterior becomes

$$p(\mathbf{m}_0|\mathbf{m}_1) = \mathcal{C}(\mathbf{m}_0|\hat{\mathbf{m}}_0) \quad \text{and} \quad p(\mathbf{m}_{t-1}|\mathbf{m}_t) = \mathcal{C}(\mathbf{m}_{t-1}|\boldsymbol{\theta}_{\text{post}}(\mathbf{m}_t, \hat{\mathbf{m}}_0))\,. \tag{5}$$

We use $1,000$ time steps for the diffusion process and adopt a linearly increasing sequence for β_t ranging from $\beta(t=0) = 10^{-4}$ to $\beta(t=1000) = 5\cdot 10^{-3}$.

Value model

We are interested in modeling the *conditional* distribution $p(\mathbf{w}|\mathbf{m})$, given N samples $\{\mathbf{m}, \mathbf{w}\}^N$, where $\mathbf{m}$ is the recipe mask and $\mathbf{w}$ is the vector of ingredient weights. The conditioning on $\mathbf{m}$ is not explicitly shown in the following derivations for simplicity, but it is implied unless otherwise stated. Score-based generative models are another class of diffusion models where the noising and denoising processes are performed using stochastic differential equations. Here, for the noising process,

$$\mathrm{d}\mathbf{w}_t = f(\mathbf{w}_t, t)\mathrm{d}t + g(t)\mathrm{d}B_t\,, \tag{6}$$

and for the denoising process,

$$\mathrm{d}\mathbf{w}_t = [g^2(t)\nabla_w \log p_t(\mathbf{w}) - f(\mathbf{w}_t, t)]\mathrm{d}t + g(t)\mathrm{d}\tilde{B}_t\,, \tag{7}$$

where B_t is K-dimensional Brownian motion, and $\tilde{B}_t$ is its time reverse, and f and g are functions that calculate the drift and diffusion processes [10, 11]. While there are a variety of options for the two stochastic differential equations, we use the Ornstein-Uhlenbeck process because of its simplicity [10, 12]. Scaling the stochastic differential equations results in the following set, for the noising process,

$$\mathrm{d}\mathbf{w}_t = -\frac{1}{2}\beta_t\mathbf{w}_t\mathrm{d}t + \sqrt{\beta_t}\mathrm{d}\mathbf{B}_t \tag{8}$$

and for the denoising process,

$$\mathrm{d}\mathbf{w}_t = \frac{1}{2}\beta(t)\mathbf{w}_t\mathrm{d}t + \beta(t)\nabla\log p_t(\mathbf{w}_t)\mathrm{d}t + \sqrt{t\beta(t)}\mathrm{d}\mathbf{B}_t\,, \tag{9}$$

with $\beta(t) = \beta_{\min} + t(\beta_{\max} - \beta_{\min})$. This results in the following simple expression for the conditional distribution,

$$p(\mathbf{w}_t|\mathbf{w}_0) = \mathcal{N}(\mathbf{w}_t|\mu(t)\mathbf{w}_0, \sigma(t)\mathbf{I}) \tag{10}$$

with mean $\mu(t)$ and variance $\sigma(t)$ given as

$$\mu(t) = \exp(-\alpha(t)/2) \quad \text{and} \quad \sigma(t) = 1-\exp(-\alpha(t)) \quad \text{with} \quad \alpha(t) = \int_0^t \beta(s)\mathrm{d}s\,. \tag{11}$$

The expression $\nabla_w \log p_t(\mathbf{w})$ is known as the *score function*. We approximate the score function by a neural network s parametrized by parameters θ, as $s_{\boldsymbol{\theta}}(\boldsymbol{w}, t) \approx \nabla_{\mathbf{w}} \log p_t(\boldsymbol{w})$ for the generative model. As a reminder, $\mathbf{x}$ is conditioned on the mask $\mathbf{m}$. When including this explicitly, the neural network approximation takes the form $s_{\boldsymbol{\theta}}(\mathbf{w}, t, \mathbf{m}) \approx \nabla_{\mathbf{w}} p_t(\mathbf{w}|\mathbf{m})$. To train the neural network, we minimize the following loss

$$L(\boldsymbol{\theta}) = \int_0^T \mathbb{E}_{p_t}\left[||\nabla_{\mathbf{w}} \log p_t(\mathbf{w}) - s_{\boldsymbol{\theta}}(\boldsymbol{w}, t)||^2\right], \tag{12}$$

where $p_t(\mathbf{w})$ is approximated using the data [10],

$$\hat{p}_t(\mathbf{w}) = \frac{1}{N} \sum_{i=1}^{N} p(\mathbf{w}|\mathbf{w}_0^i). \tag{13}$$

We use 1000 steps for the noising and denoising processes and select scaling parameters $\beta_{\text{min}} = 0.001$ and $\beta_{\text{max}} = 3$ [13].

S.1.3. Training and validation

The *mask model* is constructed using a neural network with an embeddings layer with 1000 embeddings for the time variable, and 3 fully connected layers of 512 neurons each for predicting the logits of the mask model. The logits are then used with the softmax function to produce the raw probabilities. We use minibatching with $n = 1000$ for training and train with a learning rate of $5 \cdot 10^{-4}$ using the Adam optimization algorithm for 100,000 epochs.

The *value model* is constructed using a feed-forward neural network with 4 hidden layers of 256 neurons each. The vectors for $\mathbf{x}, t$ and $\mathbf{m}$ are concatenated to form the input for $s_{\boldsymbol{\theta}}$. We use a batch size of 400 and a learning rate of $1 \cdot 10^{-3}$ with the Adam optimization algorithm and train for 20,000 epochs. The training data is split 80% for training and 20% for validation. Training is conducted on Nvidia H100 and L40S GPUs.

S.1.4. Rediscovering the Big Mac®

The original Big Mac® weighs 210 g and has 563 kcal. Since its recipe is proprietary, we approximate it using open-source recreations [14–17]. We identify the common ingredients at the intersection of the four masks,

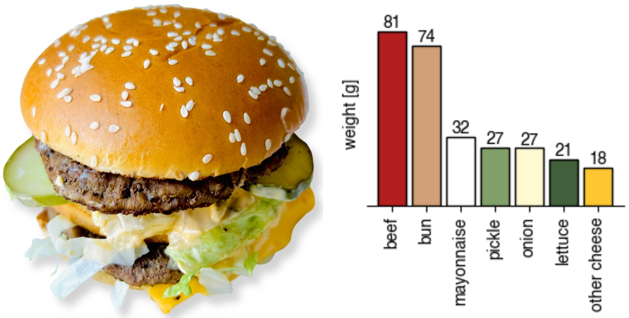

Supplementary Figure 3 Classic Big Mac® as a benchmark for rediscovery. Since the original recipe of the Big Mac® (left) is proprietary, we use online recipes to estimate its ingredients and weights as 81 g ground beef, 74 g bun, 32 g mayonnaise, 27 g pickle, 27 g onion, 21 g lettuce, and 18 g cheese (right).

and average their quantities after normalizing by calories. The resulting Big Mac® recipe for a 500 kcal serving consists of seven ingredients with the following weights: 81 g ground beef, 74 g bun, 32 g mayonnaise, 27 g pickle, 27 g onion, 21 g lettuce, and 18 g cheese (Fig. 1b main manuscript, Supplementary Fig. 3).

S.1.5. Substantial Difference Score

To quantify the proximity between recipes and grouping similar recipes we introduce the Substantial Difference Score (SDS). We define the substantial difference between two recipes r_1 and r_2 as the sum of the binary distance d_i over all ingredients in the database,

$$\mathsf{SDS}(r_1, r_2) = \sum_{i=1}^{n_{\text{ing}}} d_i(r_1, r_2), \quad \text{with} \quad d_i(r_1, r_2) = \begin{cases} 1 & \text{if } r_{1i} + r_{2i} \neq 0 \text{ and } r_{1i} \cdot r_{2i} = 0 \\ 1 & \text{if } \max(r_{1i}, r_{2i}) / \min(r_{1i}, r_{2i}) \geq 2 \\ 0 & \text{otherwise.} \end{cases} \tag{14}$$

The substantial difference score is zero, $\mathsf{SDS} = 0$, if two recipes share the same ingredients and the ratios of their quantities never exceed two. Two recipes r_1 and r_2 with a substantial difference score of zero are called repeats or a match. During rediscovery, we use the substantial difference score of zero, $\mathsf{SDS} = 0$, to identify a match between an AI-generated recipe and a human-designed recipe. During discovery, we use values larger than zero, $\mathsf{SDS} > 0$, to quantify the novelty of an AI-generated recipe compared to the human-designed recipes in the training set. The Delicious Burger 1 of the main manuscript, is the most repeated recipe with $\mathsf{SDS} = 3$ (Fig. 2c,f, main manuscript). It contains 151 g ground beef, 79 g tomato, 75 g dill pickle, 47 g yellow onion, 30 g brioche bun, 19 g mayonnaise, 12 g american cheese, 7 g lettuce, 7 g ketchup, and 1 g salt. The Delicious Burger 2 of the main manuscript, is the most repeated recipe with $\mathsf{SDS} = 6$ (Fig. 2d,g, main manuscript). It contains 62.0 g ground beef, 35.5 g bun, 28.0 g sliced tomato, 12.5 g remoulade sauce, 8.0 g Gruyère cheese, 3.5 g yellow onion, 2.0 g Worcestershire sauce, 1.0 g brown sugar, 0.5 g salt, 0.2 g garlic, and 0.2 g fresh thyme leaves.

S.1.6. Environmental Impact Score

Life cycle assessments estimate the total environmental impact of agricultural products across their production and distribution chains by surveying producers worldwide [18]. Here we quantify sustainability using the environmental database [19], a meta-analysis that harmonizes data from $n = 570$ studies. As this database does not include mushrooms, we supplement it with the averaged land-use data for cultivated mushrooms from the United States Department of Agriculture [20], and freshwater eutrophication potential, scarcity-weighted water use, and greenhouse gas emissions from three European countries [21]. To enable optimization and comparison, we condense environmental impacts into a single Environmental Impact Score (EIS) [22]. We compute EIS by normalizing and averaging impacts across four categories: land use (LU), aquatic eutrophication potential (EU), scarcity-weighted water use (WU), and greenhouse gas emissions (GHG) as

$$\mathsf{EIS} = \frac{1}{4} \left[\frac{\mathsf{LU}}{\mathsf{LU}_{\max}} + \frac{\mathsf{EU}}{\mathsf{EU}_{\max}} + \frac{\mathsf{WU}}{\mathsf{WU}_{\max}} + \frac{\mathsf{GHG}}{\mathsf{GHG}_{\max}} \right] \tag{15}$$

where the values of $\mathsf{LU}_{\max}$, $\mathsf{EU}_{\max}$, $\mathsf{WU}_{\max}$, and $\mathsf{GHG}_{\max}$ were directly obtained from [22] as 62.2 m^2, 20.2 kg $CO2$ equivalents, 174 g PO_4 equivalents, and 43,600 L. From this information, we calculate the environmental impact score for each of the ingredients in this study (Supplementary Fig. 4). We quantify the environmental impact of a recipe by summing the ingredient-level environmental impact score contributions, weighted by ingredient quantities. We manually match the ingredients from in our model (Supplementary Table 2) to one of the categories in the environmental database (Supplementary Table 3) [19]. Most ingredients in this study are whole agricultural products; however, a small number of processed ingredients, such as condiments, are composed of multiple components. Given the lack of consistent environmental data and their negligible contribution by mass, we exclude condiments, bouillons, broths, yeast, and vinegar from the environmental impact calculations. Environmentally sustainable burgers were identified by finding recipes that are repeated often in a given range of EIS scores. For example, we can generate burger recipes with $0.1 \leq \mathsf{EIS} < 0.2$ and $0.2 \leq \mathsf{EIS} < 0.3$ (Supplementary Fig. 5a,b). The Sustainable Burger 1 of the main manuscript, a plain mushroom burger with an environmental impact sore of 0.06, is the most repeated recipe with $\mathsf{EIS} < 0.1$. (Fig. 3c,f, main manuscript). It contains 226 g portobello mushroom 78 g mayonnaise, 46 g brioche bun, 21 g arugula, 9 g spicy brown mustard, 8 g extra virgin olive oil, 3 g rosemary, 2 g fresh garlic, and 1 g kosher salt. The Sustainable Burger 2 of the main manuscript, a beef-mushroom blend with an environmental impact sore of 0.51, is the most repeated recipe that contains both beef and mushroom (Fig. 3d,g, main manuscript).

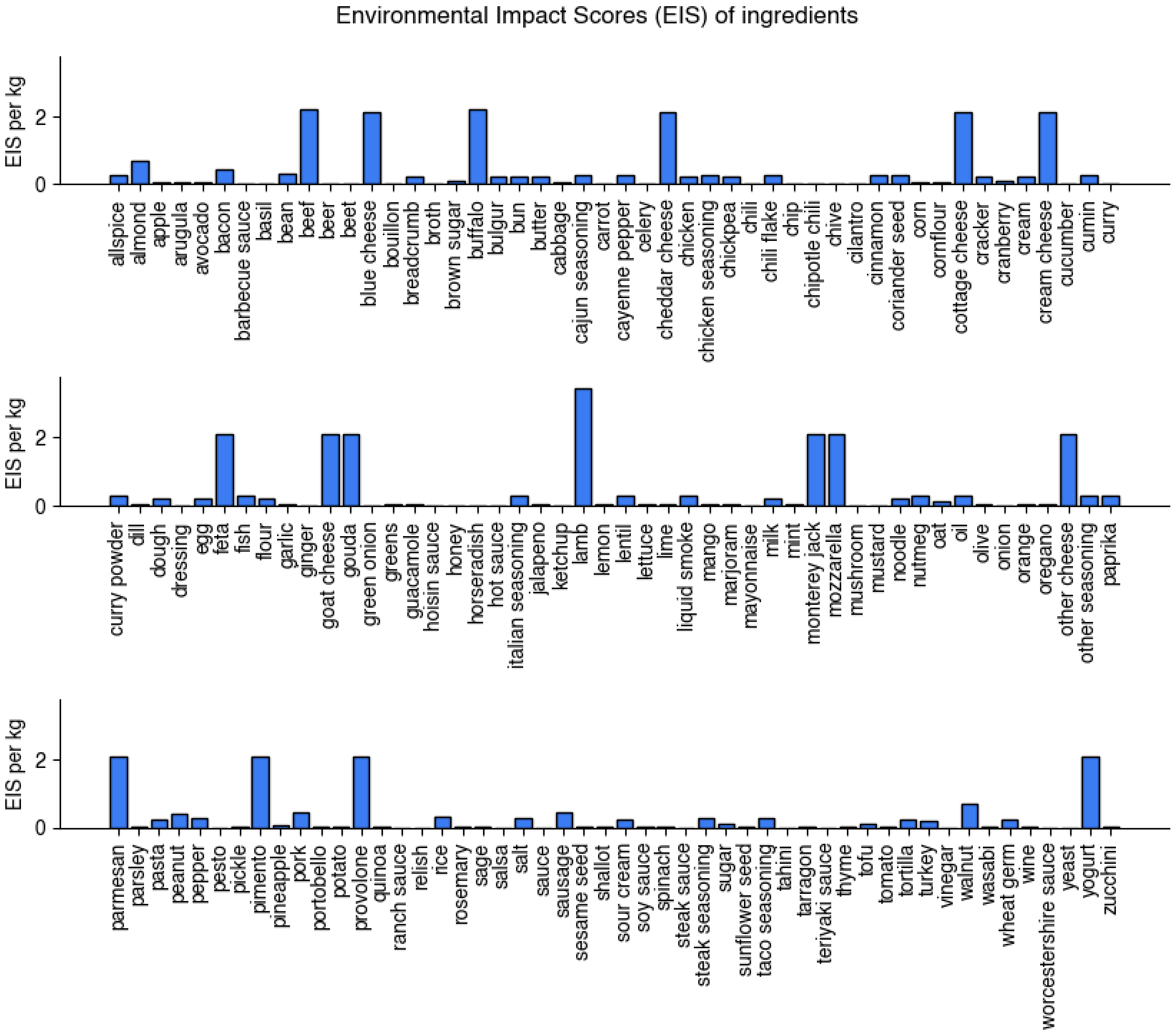

Supplementary Figure 4 **Environmental impact scores of ingredients.** Reported environmental impact scores measure the collective impact per 1 kg for each of the 146 ingredients in our database across four categories: land use, aquatic eutrophication potential, scarcity-weighted water use, and greenhouse gas emissions.

It contains 70 g ground beef, 42 g brioche bun, 39 g cheddar cheese, 19 g bacon, 17 g ketchup, 14 g crimini mushroom, 9 g yellow onion, 1 g black pepper, and 1 g kosher salt.

S.1.7. Healthy Eating Index

Nutritional profiling models quantify the nutritional quality of foods and diets by comparing nutrient and food-group composition against dietary guidelines. Common nutritional profiling models include the Healthy Eating Index (HEI) [23], the Nutri-Score [24], and the Health Star Rating [25]. The Healthy Eating Index was developed by the U.S. Department of Agriculture to assess alignment with the Dietary Guidelines for Americans [26] on a 0–100 scale, with higher scores indicating closer adherence. It primarily emphasizes food-group adequacy, such as fruits, vegetables, whole grains, and dairy, rather than individual nutrients, consistent with global trends toward food-based dietary guidelines [27]. In contrast, other nutritional profiling models explicitly emphasize nutrients of concern, including protein, fiber, potassium, calcium, iron, and vitamin D [28–30], and, in some cases, bioactive compounds such as flavonoids [31]. Here we use the healthy eating index as the primary metric to evaluate the nutritional quality of our generated burger recipes. We

Supplementary Table 3 Mapping of our 146 burger ingredients onto environmental database LCA categories. To calculate the environmental impact score, we map each the 146 ingredients from in our model (Supplementary Table 2) to one of the LCA categories in the environmental database.

LCA Category	Burger Ingredients
Wheat & Rye (Bread)	breadcrumb, bulgur, bun, cracker, dough, flour, noodle, pasta, tortilla, wheat germ
Maize (Meal)	corn, cornflour
Barley (Beer)	beer
Oatmeal	oat
Rice	rice
Potatoes	chip, potato
Sugar	brown sugar, sugar
Other Pulses	bean, lentil
Peas	chickpea
Nuts	almond, walnut
Groundnuts	peanut
Soymilk	soy sauce
Tofu	tofu
Oil	oil
Tomatoes	tomato
Onions & Leeks	green onion, chive, onion, shallot
Root Vegetables	beet, carrot, ginger, horseradish, wasabi
Brassicas	arugula, cabbage, lettuce
Other Vegetables	basil, celery, chili, chipotle chili, cilantro, cucumber, dill, garlic, greens, jalapeno, marjoram, mint, oregano, parsley, pickle, quinoa, rosemary, sage, sesame seed, spinach, sunflower seed, tarragon, thyme, zucchini
Citrus Fruit	lemon, lime, orange
Apples	apple
Berries & Grapes	cranberry
Wine	wine
Other Fruit	avocado, mango, guacamole, olive, pineapple
Beef	beef
Lamb & Mutton	lamb
Pig Meat	bacon, pork, sausage
Poultry Meat	chicken, turkey
Milk	milk
Cheese	blue cheese, cheddar cheese, cottage cheese, cream cheese, feta, goat cheese, gouda, monterey jack, mozzarella, other cheese, parmesan, pimento, provolone, yogurt
Eggs	egg
Fish (farmed)	fish
Sweeteners & Honey	honey
Stimulants & Spices Misc.	allspice, cajun seasoning, cayenne pepper, chicken seasoning, chili flake, cinnamon, coriander seed, cumin, curry powder, italian seasoning, liquid smoke, nutmeg, other seasoning, paprika, pepper, salt, steak seasoning, taco seasoning
Buffalo	buffalo
Butter, Cream & Ghee	butter, cream, sour cream
Mushrooms	portobello, mushroom
Condiments & Others	barbecue sauce, curry, dressing, hoisin sauce, hot sauce, ketchup, mayonnaise, mustard, pesto, ranch sauce, relish, salsa, sauce, steak sauce, tahini, teriyaki sauce, worcestershire sauce, bouillon, broth, yeast, vinegar

sample a large number of recipes from the generative model and identify representative nutritious burgers by selecting the most frequently occurring recipe among those in the 90th and 95th percentiles of the healthy eating index distribution. Selecting highly repeated samples ensures that the resulting recipes correspond to regions of high probability under the generative model and are therefore likely to be palatable and popular. The Nutritious Burger of the main manuscript, a bean-based burger with a healthy eating index of 63.12, is the most repeated recipe in the 95th percentile of the healthy eating index (Fig. 4d, main manuscript). It contains 71 g canned kidney beans, 50 g brioche bun, 15 g rolled oats, 10 g egg, 4 g corn flour, 4 g extra virgin olive oil, 2 g jalapeño, 2 g cilantro, 2 g white onion, 0.8 g kosher salt 0.2 g cumin, 0.2 g black pepper, and 0.05 g

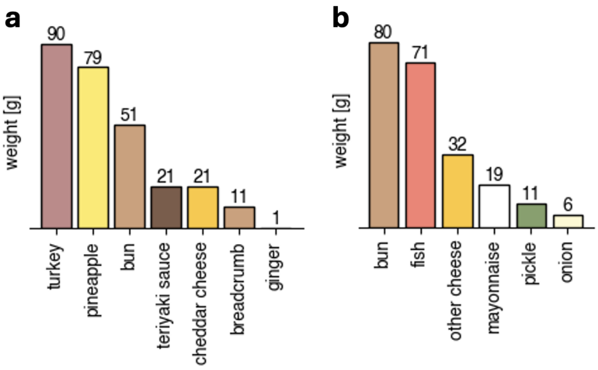

Supplementary Figure 5 **Alternative sustainable burgers.** Most repeated recipes with an environmental impact score of $0.1 \leq$ EIS < 0.2 (a) and $0.2 \leq$ EIS < 0.3 (b), ranking between the Sustainable Burger 1 with EIS $= 0.03$ and Sustainable Burger 2 with EIS $= 0.51$ of the main manuscript.

Mexican oregano. For comparison, is the most repeated recipe in the 90th percentile of the healthy eating index is a bean-corn blend with different ingredients and nutritional profile (Supplementary Fig. 6a,b).

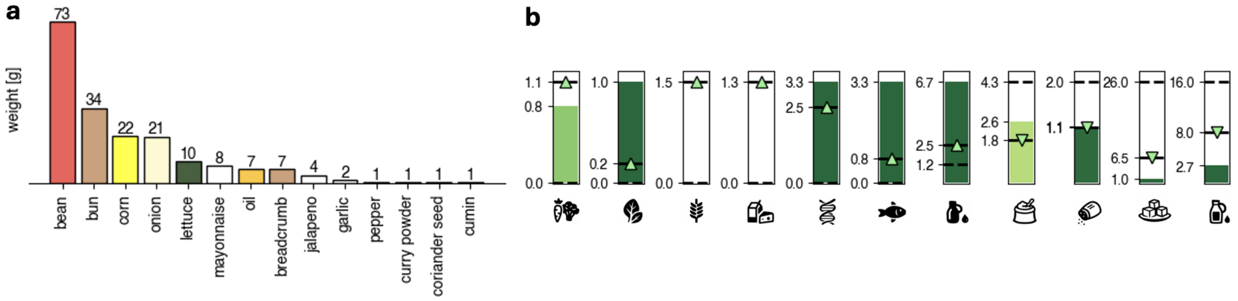

Supplementary Figure 6 **Alternative nutritious burger.** Most repeated recipe in the 90th percentile of the healthy eating index (HEI), a bean-corn blend with ingredients (a) and nutritional profile (b).

In addition, we use the balanced hybrid nutrient density score (bHNDS) [28, 32], a combined food-group- and nutrient-based nutritional profiling model, to generate alternative nutritious burger formulations. For comparison, we generated the 95th and 90th percentile nutritious burger recipes by balanced hybrid nutrient density score (Supplementary Fig. 7a,b).

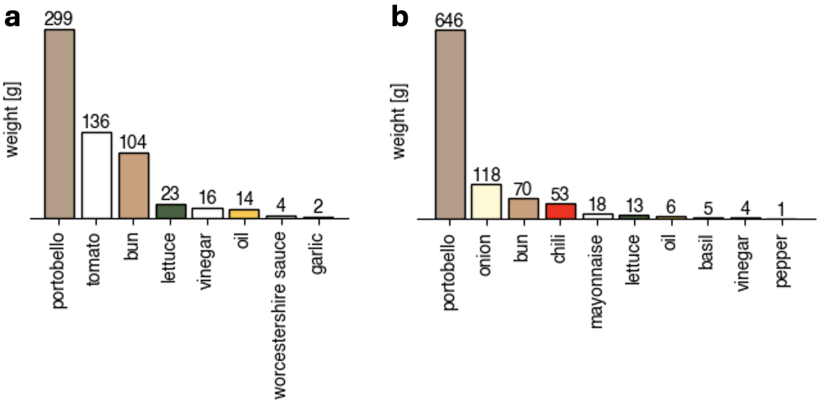

Supplementary Figure 7 **Alternative nutritious burgers.** Most repeated recipes in the 95th percentile (a), and 90th percentile (b) by balanced hybrid nutrient density score (bHNDS).

Personalized nutrition

Nutritional requirements vary substantially across individuals as a function of age, sex, body composition, physical activity, and physiological status. For example, dietary reference intakes for iron in adult women aged 19–50 years are more than twice those for men of the same age group [33]. To account for such heterogeneity, we adopt a *personalized nutrient profiling model* [34] that enables the generation of recipes tailored to individual nutritional requirements. We compute a personalized nutrition score on a 0–100 scale based on individual characteristics, including age, sex, body weight, height, and physical activity level. We derive recommended intake ranges from dietary reference intakes and acceptable macronutrient distribution ranges [33, 35], together with World Health Organization guidelines on upper intake limits for sodium, free sugars, and saturated fats [36–38]. These recommendations jointly define nutrient-specific target ranges that are aggregated into a single *personalized nutrition score* for each burger. Using this framework, we generate personalized burger recipes for four representative demographic profiles: a 30-year-old sedentary male of 80 kg and 180 cm, a 30-year-old sedentary female of 60 kg and 160 cm, a very active 15-year-old male of 55 kg and 160 cm, and a moderately active 70-year-old female of 70 kg and 170 cm (Supplementary Fig. 8).

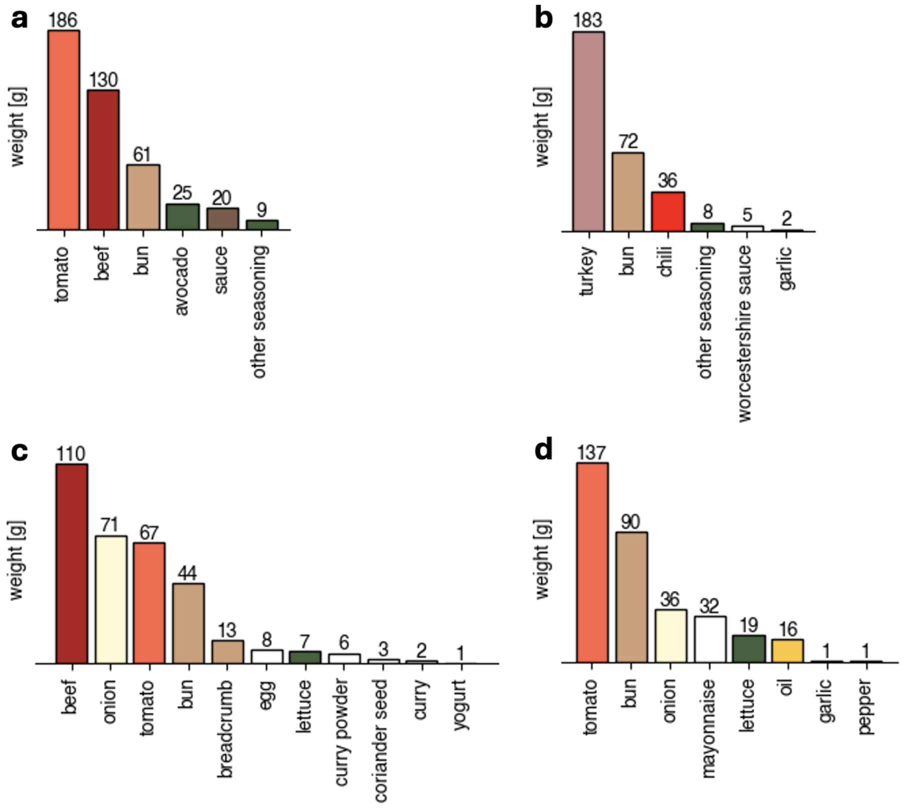

Supplementary Figure 8 Personalized nutritious burgers. Personalized recipes for a sedentary 30 year old male weighing 80 kg with a 180 cm height (a), a sedentary 30 year old female weighing 60 kg with a 160 cm height (b), a very active 15 year old male weighing 55 kg with a 160 cm height (c), and a moderately active 70 year old female weighing 70 kg with a 170 cm height (d).

S.2. Sensory evaluation

We enrolled $n = 101$ voluntary participants from the general population for a blind sensory evaluation of all six burgers at an active restaurant in San Francisco, CA (Supplementary Fig. 9). The study was performed in accordance with Stanford University Institutional Review Board guidelines. We asked the participants to answer seven question about their background, and then, for each burger, rank overall liking, flavor, and texture on a 7-point Likert scale, and check-all-that-apply for 12 flavor attributes and 15 texture attributes.

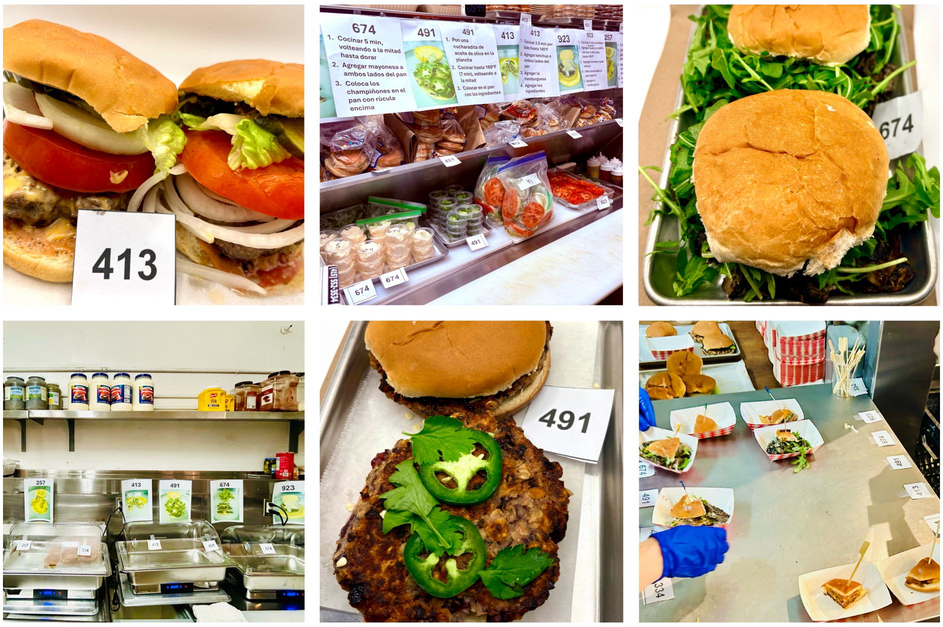

Supplementary Figure 9 **Sensory evaluation.** Voluntary participants from the general population enrolled in a blind sensory evaluation of all six burgers in an active restaurant. Snapshots during the preparation of all six burgers and of Sustainable Burger 1 labeled as 413, Delicious Burger 1 labeled as 674, and Nutritious Burger labeled as 491, prior to sampling.

S.2.1. Participant information

What is your age?

Select one

- Less than 18
- 18-25
- 26-35
- 36-45
- 46-55
- Greater than 55

Which gender do you identify as?

Select one

- Male
- Female
- Non-binary
- Prefer not to say

What is the highest degree or level of education you have completed?

Select one

- Some high school
- High school
- Some college
- Bachelor's degree
- Master's degree
- Ph.D. or higher
- Trade school

How often do you eat burgers of any kind?

Select one

- Everyday
- 2-3 times a week
- Once a week
- 2-3 times a month
- Once every 1-2 months
- 4-5 times a year
- 2-3 times a year
- Never or rarely

What is your dietary preference?

Select one

- Vegan
- Vegetarian
- Prescatarian
- Flexitarian (i.e. eats meat occasionally)
- Omnivore (i.e. eats meat regularly)

Where do you shop regularly for groceries?

Select all that apply

- Natural grocers (e.g. Whole Foods)
- Discount grocers (e.g. Trader Joe's, Lidl, Aldi)
- Conventional grocers (e.g. Stop & Shop, Safeway)
- Big box retailers (e.g. Target, Walmart)
- Grocery delivery services (e.g. Instacart, Amazon fresh)
- Meal kits (e.g. Blue Apron)
- Bulk grocers (e.g. Costco, Sam's Club)
- Small neighborhood grocers
- Directly from the brand/manufacturer's website

What matters most to you when deciding what to eat?

Select all that apply

- Health
- Price
- Taste
- Convenience
- Familiarity (what I'm used to)
- Environment
- Animal welfare

S.2.2. Sensory feedback

How would you rate your overall liking of Burger X?

Select one

- Like very much
- Like
- Like somewhat
- Neither like nor dislike
- Dislike somewhat
- Dislike
- Dislike very much

How would you rate the flavor of Burger X?

Select one

- Like very much
- Like
- Like somewhat
- Neither like nor dislike
- Dislike somewhat
- Dislike
- Dislike very much

Please check all the words or phrases that describe the flavor of Burger X.

Select all that apply

- Meaty
- Weird Aftertaste
- Off-flavor
- Bland
- Earthy / Soil
- Smoky
- Strong
- Sweet
- Salty
- Fatty
- Savory
- Good aftertaste

How would you rate the texture of Burger X?

Select one

- Like very much
- Like
- Like somewhat
- Neither like nor dislike
- Dislike somewhat
- Dislike
- Dislike very much

Please check all the words or phrases that describe the texture of Burger X.

Select all that apply

- Chewy
- Crispy / Crunchy

- Crumbly / Grainy
- Firm / Hard
- Soft / Mushy
- Holds together
- Moist
- Dry
- Tough
- Fatty
- Fibrous / Stringy
- Brittle
- Gummy
- Springy
- Sticky

S.2.3. Population demographics

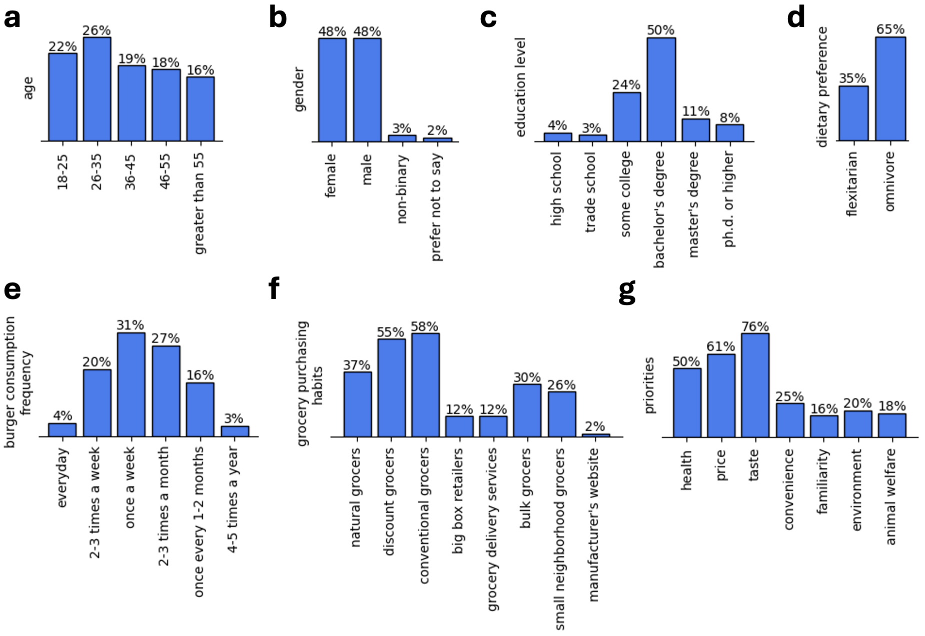

Supplementary Figure 10 Population demographics. Overview of the demographics and food preferences of the survey respondents. Participant age, gender, education, dietary preference, burger consumption frequency, grocery purchasing habits, and priorities from the responses to the first seven survey questions.

The $n = 101$ study participants represent a balanced cross section of the general population (Supplementary Fig. 10). Of the $n = 101$ participants, 47.5% were male, 47.5% female, 3% non-binary, and 2% prefer not to say. 22% were 18-25 years old, 26% are 26-35, 19% are 36-45, 18% are 46-55, and 16% are older than 55. 65% were omnivores and 35% were flexitarians. Their highest degree of education was 4% high school degree, 24% college, 50% batchelor's, 11% master's, 8% Ph.D. or higher, and 3% trade school. 4% eat burgers every day, 20% 2-3 times per week, 31% once a week, 27% 2-3 times per month, 16% every 1-2 months, and 3% 4-5 times per year. For grocery purchasing habits, 37% preferred natural grocers, 55% discount grocers, 58% conventional grocers, 12% big box retailers, 12% grocery delivery services, 30% bulk grocers, 26% small neighborhood grocers, and 2% manufacturer's websites. For purchasing priorities, 50% listed health, 61% price, 76% taste, 25% convenience, 16% familiarity, 20% environment, and 18% animal welfare.

S.3. Guidelines for Recipe Development

This document was handed to an Executive Chef. It contains the AI-generated ingredient lists and their corresponding quantities, and describes how the lists should be interpreted.

S.3.1. Recipes

This study includes 5 burgers. The ingredients and their quantities for the 5 burgers are given below. Guidelines for the Chefs follow in the next section.

Delicious Burger 1

- beef 151 g
- bun 30 g
- ketchup 7 g
- lettuce 7 g
- mayonnaise 19 g
- onion 47 g
- other cheese 12 g
- pickle 75 g
- tomato 79 g

Delicious Burger 2

- beef 124 g
- brown sugar 2 g
- bun 71 g
- garlic 0.4 g
- onion 7 g
- other cheese 16 g
- other sauce 25 g
- thyme 0.3 g
- tomato 56 g
- worcestershire sauce 4 g

Sustainable Burger 1

- arugula 21 g
- bun 46 g
- garlic 2 g
- mayonnaise 78 g
- mustard 9 g
- oil 8 g
- portobello 226 g
- rosemary 3 g

Sustainable Burger 2

- bacon 19 g
- beef 70 g
- bun 42 g
- cheddar cheese 39 g
- ketchup 17 g
- mushroom 14 g
- onion 9 g
- black pepper 1 g

Nutritious Burger

- bean 71 g
- bun 50 g
- cilantro 2 g
- cornflour 4 g
- cumin 0.2 g
- egg 10 g
- jalapeno 2 g
- oat 15 g
- oil 4 g
- onion 2 g
- oregano 0.05 g
- black pepper 0.2 g

S.3.2. Guidelines

1. The lists in the previous section show the ingredient amounts that should be used in each burger. The chefs should develop recipes with these ingredients and amounts, without adding or removing any ingredients.
2. The ingredient lists only show the ingredients, but all processing steps are to be decided by the chefs.
3. All the ingredients are listed in singular words, e.g., oat instead of oats, onion instead of onions, etc. This is simply for convenience.
4. The chefs should decide which parts of the ingredient lists should be used in the patty, and which parts should be layered on top. This could include dividing some ingredients into two or more groups. For example, if the recipe mentions onion, the chef could decide to mince some of it and add to the patty, and slice some for layering on top of the patty. In such cases, we ask the chefs to record the weights of the parts.
5. The amounts given in each recipe are adjusted such that each ingredient list adds up to 500 calories. The chefs are free to adjust the quantities such that the ingredients make up one burger instead, as long as the ratio of the ingredients remains unchanged. For example, if the chef believes that the ingredients listed for a burger would make 2 burgers, they are free to divide the amounts of the ingredients by 2 as long as this is done for each ingredient.
6. The weights are generally meant to be dry and uncooked weights. If the chef believes that the weight listed for an ingredient is too high/low for it to be the dry/uncooked weight, e.g., beans, please contact us know for further clarification.
7. If the amount for a herb or spice is extremely low, the chef can reinterpret it as a really small amount, e.g., 0.05 g of oregano can be interpreted as a couple of leaves of oregano.
8. salt: the ingredient lists do not explicitly list salt. The chefs should adjust salt to taste, and record the amount they used.
9. All of the listed ingredients are meant to be used in preparing the burgers, but not all of them have to stay on the burger. Examples:
   - Vegetables and fruits have peels and stems that can be removed after weighing.
   - If the recipe lists oil, and if the chef decides that the oil is best used for frying or sauteing, the oil may remain in the pan after sauteing
   - Some herbs like rosemary may be used to add aroma to the rest of the ingredients and disposed afterwards.
10. Some of the recipes may result in overflowing burgers, this is acceptable but the chefs are still kindly asked to try their best in preparing the burgers in a way that is presentable, such that the burger has a structural integrity.
11. The ingredient lists do not mention specific details like variety of the ingredients. The chefs are free to make their own decisions about this. Examples:
    - Beef may be interpreted as intact meat or gound beef, depending on what the chef believes is the best choice for that particular recipe.
    - Vegetables can be any variety that the chef prefers, e.g., red onions, white onions, etc., but not green onions, which are a different product.

12. Other cheese includes any cheese that is not one of these: blue cheese, cheddar cheese, cottage cheese, cream cheese, feta, goat cheese, gouda, monterey, monterey jack, mozzarella, parmesan, provolone. We ask the chef to select a cheese that they think best fits the rest of the ingredients.
13. Other sauce includes any sauce that is not one of these: barbecue sauce, buffalo sauce, cream, curry sauce, hoisin sauce, honey, hot pepper sauces, ketchup, mustard, pesto, ranch, relish, salsa, soy sauce, steak sauce, teriyaki sauce, vinegar, wasabi, Worcestershire sauce. We ask the chef to select a sauce that they think best fits the rest of the ingredients.
14. In Sustainable Burger 2, we ask that the chef combine the meat and mushrooms together to make the patty. This way the burger is more aligned with commercially available beef-mushroom blends.
15. We kindly ask the chefs to put their best creativity and artistic skills to work to turn these ingredient lists to the best burgers possible, and wholeheartedly thank them for their help in this study!

S.4. Recipes with ingredients and preparation instructions

This document was prepared by an Executive Chef. It contains the AI-generated ingredient lists, their quantities, and the preparation steps and photos of the assembled burgers as designed by the chef. For comparison, we also include the ingredient list of the Big Mac®, although we purchased it for the survey to provide the most authentic experience.

Big Mac®

List of ingredients

- 81 g ground beef
- 74 g bun
- 32 g mayonnaise
- 27 g pickle
- 27 g onion
- 21 g lettuce
- 18 g cheese

Purchased product

Supplementary Figure 11 shows the Big Mac®as a benchmark comparison in the sensory survey. The Big Mac®was not prepared by the chefs but purchased to provide the most authentic sensory experience.

Delicious Burger 1

List of ingredients

- 151 g ground beef (80/20)
- 30 g brioche bun, trimmed significantly to make weight
- 7 g ketchup
- 7 g lettuce, little gem
- 19 g mayo
- 47 g yellow onion, sliced rings
- 12 g American cheese, slice
- 75 g dill pickle, chips
- 79 g tomato, sliced
- 1 g kosher salt

Preparation instructions

Supplementary Figure 12 shows the Delicious Burger 1 prepared according to the following instructions: Using a ring mold slightly larger than the bun, shape the mixture into a patty, pressing it into an even layer inside the mold. Season both sides of the patty with salt. Preheat a nonstick skillet over medium heat. Lightly toast the bun in the dry pan until golden brown, then remove and set aside. Add the patty to the hot pan and

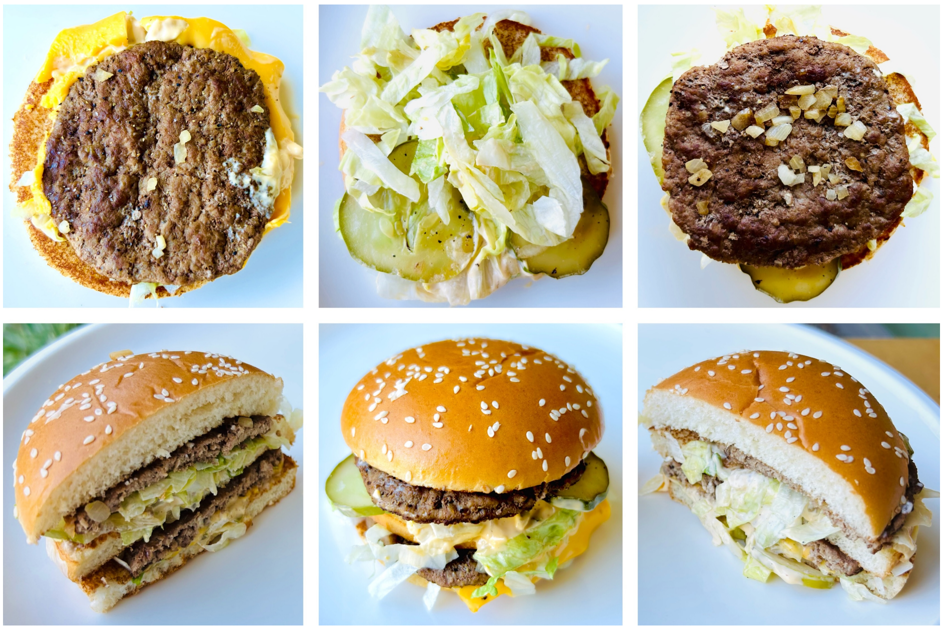

Supplementary Figure 11 Big Mac®. The Big Mac®is classic beef burger with seven main ingredients, ground beef, mayonnaise, pickle, onion, lettuce, and cheese, served in a bun.

cook for 3.5 to 4 minutes per side, adding the cheese during the last minute to allow it to melt. In a small bowl, mix the mayonnaise and ketchup until combined, then spread the sauce evenly on both sides of the bun. Assemble the burger by stacking the lettuce, tomato, pickles, and onions carefully on top of the patty.

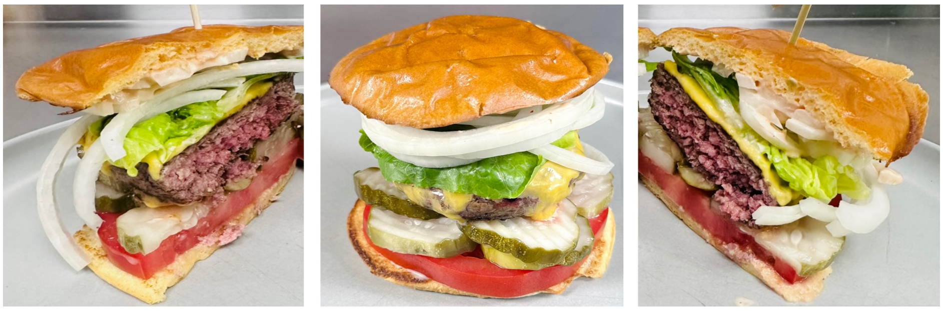

Supplementary Figure 12 Delicious Burger 1. The Delicious Burger 1 is a classical beef burger optimized for deliciousness with nine ingredients, beef, tomato, pickle, onion, mayo, cheese, lettuce and ketchup, served in a bun.

Delicious Burger 2

List of ingredients

- 62 g ground beef (80/20)
- 1 g brown sugar
- 35.5 g bun
- 0.2 garlic clove, microplaned
- 3.5 g yellow onion, shaved
- 8 g Gruyère cheese, sliced
- 12.5 g rémoulade sauce
- 0.15 g fresh thyme leaves, minced
- 28 g sliced tomato
- 2 g Worcestershire sauce

- 0.5 g kosher salt

Preparation instructions

Supplementary Figure 13 shows the Delicious Burger 2 prepared according to the following instructions: In a bowl, thoroughly mix the beef with the garlic, thyme, brown sugar, and Worcestershire sauce. Using a ring mold slightly bigger than the bun, shape the mixture into a patty pressing it into an even layer inside the mold. Preheat a nonstick pan over medium-high heat. Lightly toast the bun in the dry pan until golden brown, then remove and set aside. Season the burger patty with salt. Arrange the sliced onions on top of the patty, then place it in the hot pan onion-side down. Cook for 2 minutes, then flip the patty, add the cheese, and cook for another 2 minutes. Cover the pan with a lid to help the cheese melt. To assemble, layer the sliced tomato on the bottom bun, followed by the burger patty. Spread the remoulade sauce on the top bun, then close the burger.

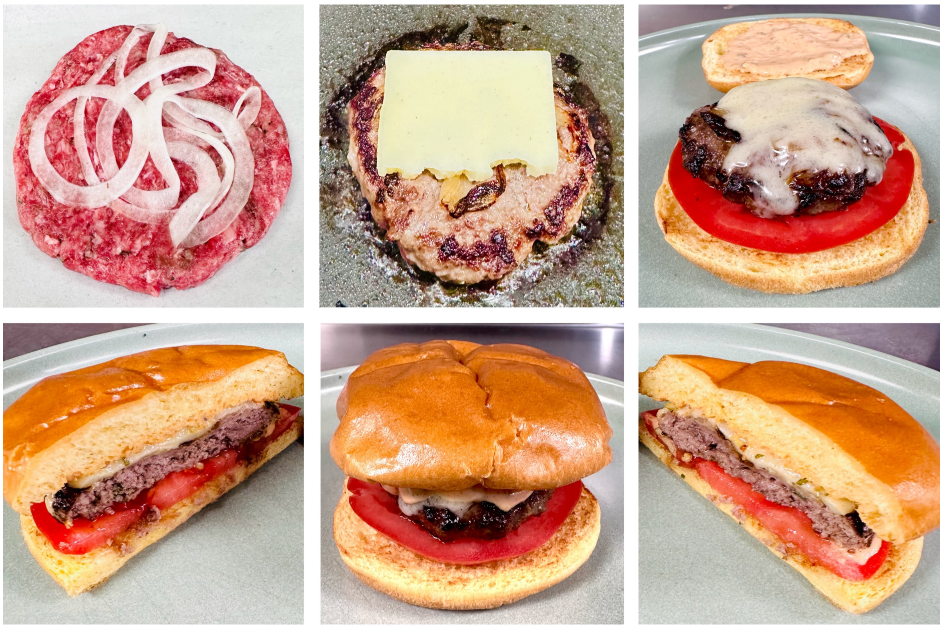

Supplementary Figure 13 Delicious Burger 2. The Delicious Burger 2 is an innovative beef burger optimized for deliciousness with ten ingredients, beef, tomato, remoulade, Gruyère cheese, onion, Worcestershire sauce, brown sugar, garlic, and thyme leaves, served in a bun.

Sustainable Burger 1

List of ingredients

- 21 g arugula
- 46 g brioche bun (trimmed to make weight)
- 2 g fresh garlic, microplaned
- 78 g mayonnaise, divided (Best Foods)
- 9 g spicy brown mustard
- 8 g extra virgin olive oil
- 226 g portobello mushroom (stems and gills removed)
- 3 g rosemary, minced
- 0.5 g kosher salt

Preparation instructions

Supplementary Figure 14 shows the Sustainable Burger 1 prepared according to the following instructions: Slice portobello into 0.5 inch thick slices across the cap. In a bowl, mix together the garlic, mustard, rosemary,

salt, and half of the mayonnaise. Add the mushrooms and fold gently to coat. Let marinate for 20 minutes. Preheat a nonstick pan over medium-high heat. Lightly toast the bun in the dry pan until golden, then remove and set aside. Add the olive oil to the same pan. Once hot, add the marinated mushroom slices and caramelize for about 2.5 minutes per side, until deeply browned. Transfer the mushrooms to a paper towel to drain any excess oil. Spread the remaining mayonnaise on both sides of the bun. Layer the mushrooms on the bottom half and top with fresh arugula.

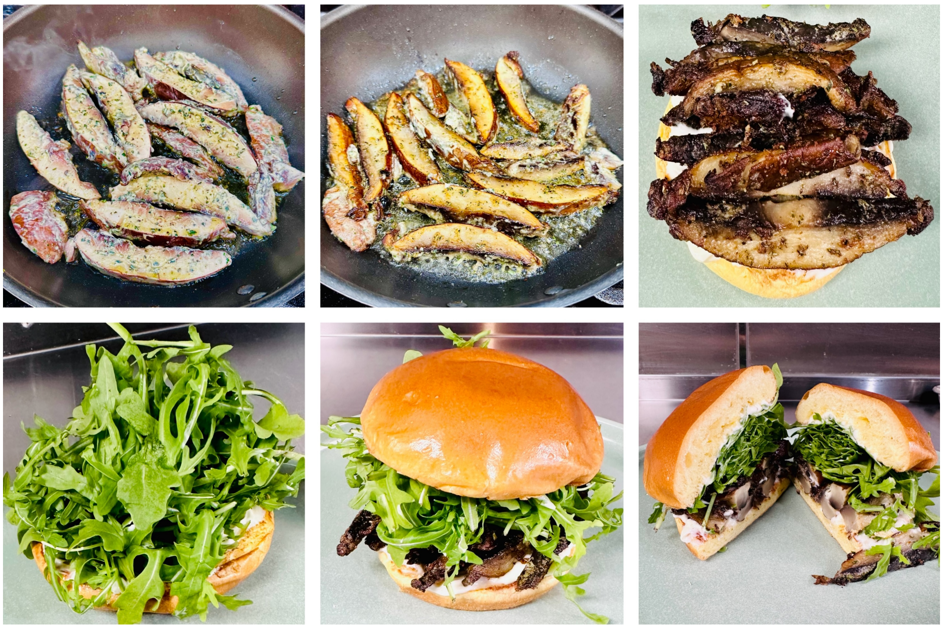

Supplementary Figure 14 **Sustainable Burger 1.** The Sustainable Burger 1 is a mushroom burger optimized for sustainability with eight ingredients, portobello mushroom, mayonnaise, arugula, mustard, oil, rosemary, and garlic, served in a bun.

Sustainable Burger 2

List of ingredients

- 19 g bacon, cut into two pieces
- 70 g ground beef (80/20)
- 42 g brioche bun, slightly trimmed to make weight
- 39 g cheddar cheese, sliced
- 17 g ketchup
- 14 g crimini mushroom, finely minced
- 9 g yellow onion, shaved
- 1 g black pepper, ground
- 0.8 g kosher salt

Preparation instructions

Supplementary Figure 15 shows the Sustainable Burger 2 prepared according to the following instructions: Combine the minced mushrooms and ground beef, folding them together until evenly mixed. Shape the mixture into a ball, then roll it in salt and pepper to coat. Preheat a nonstick skillet over medium heat. Lightly toast the bun in the dry pan until golden, then remove and set aside. Reduce the heat to medium-low, add the bacon, and cook until the fat renders and the bacon is crisp. Transfer the bacon to a paper towel–lined plate to drain, leaving the rendered fat in the pan. Increase the heat to medium-high. Place the sliced onions in the center of the skillet, then set the seasoned meat ball on top. Cover with a piece of parchment or wax paper, and firmly smash the patty with a spatula until it's slightly wider than the bun. Cook for 1.5 minutes, then flip, top with cheese, and cook for another 1.5 minutes, or until the patty

is cooked through and the cheese has melted. Spread ketchup on both sides of the toasted bun. Place the burger patty on the bottom half, layer with bacon, and top with the other half of the bun.

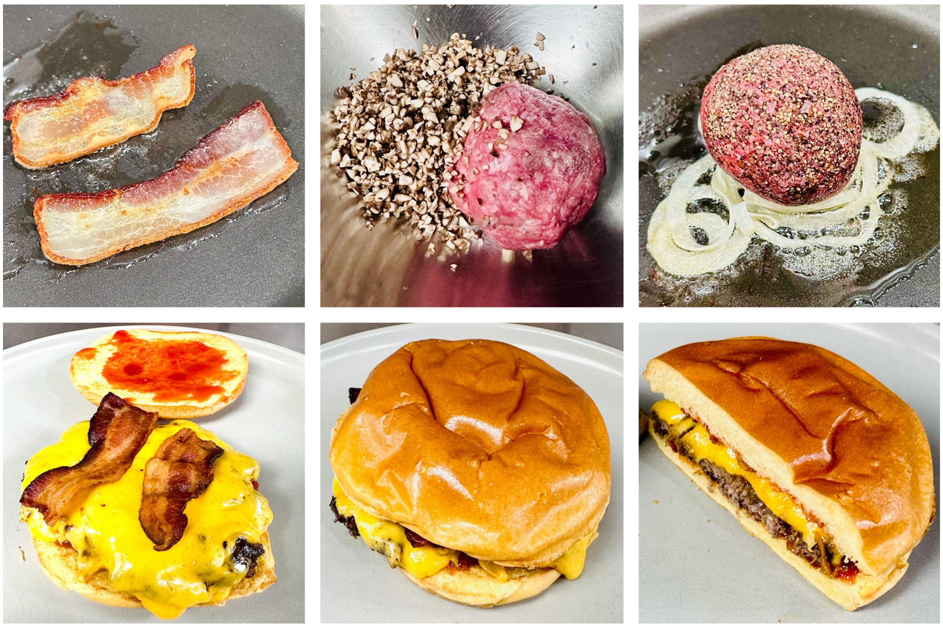

Supplementary Figure 15 **Sustainable Burger 2.** The Sustainable Burger 2 is a mushroom-beef blend with eight ingredients, ground beef, cheddar cheese, bacon, ketchup, crimini mushroom, onion, and pepper, served in a bun.

Nutritious Burger

List of ingredients

- 71 g canned kidney beans (drained and rinsed)
- 50 g brioche bun, lightly toasted
- 2 g cilantro (leaves and tender stems)
- 4 g corn flour
- 0.2 g cumin, ground
- 10 g egg (whisk one whole egg and measure out 10 g)
- 2 g jalapeño, thinly sliced rounds
- 15 g rolled oats
- 4 g extra virgin olive oil
- 2 g white onion, minced
- 0.05 g Mexican oregano, dried
- 0.2 g black pepper, ground
- 0.8 g kosher salt, divided between the patty mix and seasoning the formed patty before cooking

Preparation instructions

Supplementary Figure 16 shows the Nutritious Burger prepared according to the following instructions: Lightly mash the beans with a fork, leaving some chunks for texture. Stir in the onion, cumin, half of the salt, black pepper, and oregano. Add the oats and corn flour, mixing until evenly combined. Finally, add the egg and mix thoroughly to form a cohesive mixture. Using a ring mold the same size as your bun, shape the mixture into a patty on a piece of parchment paper. Refrigerate for 30 minutes to allow the starches to hydrate and the patty to firm up before cooking. Preheat a nonstick pan over medium-high heat and lightly toast the bun in the dry pan. Once the bun is toasted, add the olive oil to the pan. Season the chilled patty with the remaining salt and cook for about 3.5 minutes per side, until golden and heated through. Assemble the burger by placing the cooked patty on the toasted bun, then topping with the remaining cilantro and jalapeño slices.

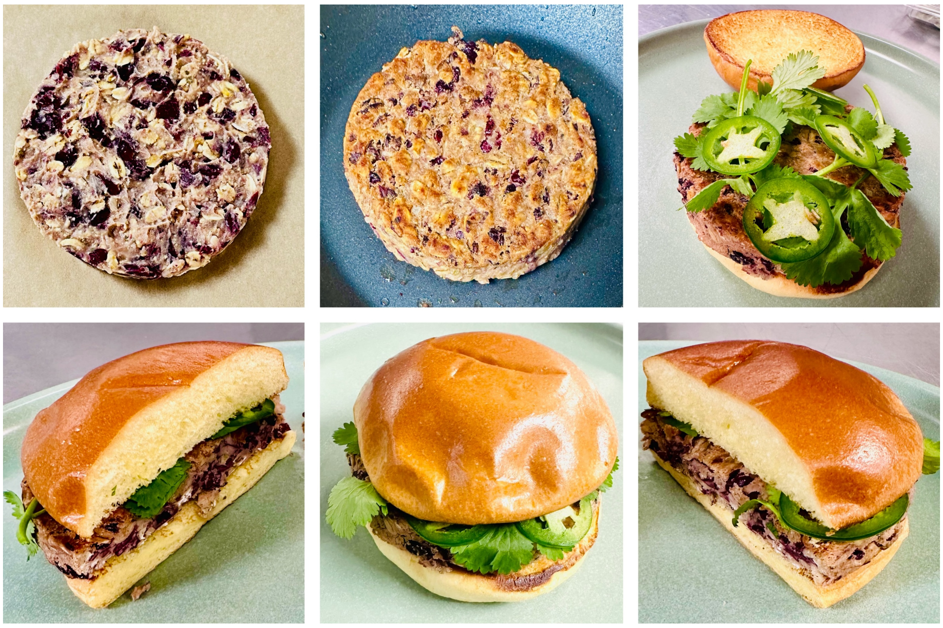

Supplementary Figure 16 Nutritious Burger. The Nutritious Burger is a bean-based burger optimized for nutrition with twelve ingredients, kidney beans, rolled oats, egg, cornflour, oil, cilantro, jalapeño, onion, cumin, pepper, and oregano, served in a bun.

References

[1] Alvin. Food.com - Recipes and Reviews (2020).

[2] Wei, Alexander. Food.com Recipes with Ingredients and Tags (2023).

[3] Jiang, A. Q. *et al.* Mistral 7B (2023). arXiv:2310.06825.

[4] Charrondiere, U. Ruth, Haytowitz, David & Stadlmayr, Barbara. FAO/INFOODS Databases (2012).

[5] Aqua-Calc. Aqua-Calc: online calculator for physical water properties. www.aqua-calc.com (2025). Accessed on December 13, 2025.

[6] kg-m3. kg/m3 All about material density. www.kg-m3.com (2025). Accessed on December 13, 2025.

[7] U.S. Department of Agriculture (USDA). *FoodData Central: Foundation Foods* (Agricultural Research Service, 2024).

[8] Hoogeboom, E., Nielsen, D., Jaini, P., Forré, P. & Welling, M. Ranzato, M., Beygelzimer, A., Dauphin, Y., Liang, P. & Vaughan, J. W. (eds) *Argmax flows and multinomial diffusion: Learning categorical distributions.* (eds Ranzato, M., Beygelzimer, A., Dauphin, Y., Liang, P. & Vaughan, J. W.) *Advances in Neural Information Processing Systems*, Vol. 34, 12454–12465 (2021).

[9] Ho, J., Jain, A. & Abbeel, P. Larochelle, H., Ranzato, M., Hadsell, R., Balcan, M. & Lin, H. (eds) *Denoising diffusion probabilistic models.* (eds Larochelle, H., Ranzato, M., Hadsell, R., Balcan, M. & Lin, H.) *Advances in Neural Information Processing Systems*, Vol. 33, 6840–6851 (2020).

[10] Taç, V., Rausch, M. K., Bilionis, I., Sahli Costabal, F. & Tepole, A. B. Generative hyperelasticity with physics-informed probabilistic diffusion fields. *Engineering with Computers* **41**, 51–69 (2024).

[11] Song, Y. *et al.* Score-based generative modeling through stochastic differential equations. *arXiv* doi:10.48550/arXiv.2011.13456 (2021).

[12] Pidstrigach, J. Koyejo, S. *et al.* (eds) *Score-based generative models detect manifolds.* (eds Koyejo, S. *et al.*) *Advances in Neural Information Processing Systems*, Vol. 35, 35852–35865 (2022).

[13] Karras, T., Aittala, M., Aila, T. & Laine, S. Koyejo, S. *et al.* (eds) *Elucidating the design space of diffusion-based generative models.* (eds Koyejo, S. *et al.*) *Advances in Neural Information Processing Systems*, Vol. 35, 26565–26577 (2022).

[14] Taylor Stinson. The Best Homemade Big Mac Recipe (2024).

[15] Theodora Kaloudis. Big Mac (2024).

[16] Krystle Smith. Big Mac Recipe (2024).

[17] Ron Dimpflmaier. Make Your Own BIG MAC.

[18] Hellweg, S. & Milà I Canals, L. Emerging approaches, challenges and opportunities in life cycle assessment. *Science* **344**, 1109–1113 (2014).

[19] Poore, J. & Nemecek, T. Reducing food's environmental impacts through producers and consumers. *Science* **360**, 987–992 (2018).

[20] Mushrooms. Tech. Rep. ISSN: 1949-1530, National Agricultural Statistics Service (NASS) (2016).

[21] Goglio, P. *et al.* An environmental assessment of Agaricus bisporus ((J.E.Lange) Imbach) mushroom production systems across Europe. *European Journal of Agronomy* **155**, 127108 (2024).

[22] Clark, M. *et al.* Estimating the environmental impacts of 57,000 food products. *Proceedings of the National Academy of Sciences* **119**, e2120584119 (2022).

[23] Krebs-Smith, S. M. *et al.* Update of the Healthy Eating Index: HEI-2015. *Journal of the Academy of Nutrition and Dietetics* **118**, 1591–1602 (2018).

[24] Julia, C., Etilé, F. & Hercberg, S. Front-of-pack Nutri-Score labelling in France: An evidence-based policy. *The Lancet Public Health* **3**, e164 (2018).

[25] Commonwealth of Australia. Health star rating system. https://www.healthstarrating.gov.au/ (2014). URL https://www.healthstarrating.gov.au/. Accessed: 2025-12-10.

[26] 2015-2020 Dietary Guidelines for Americans. Tech. Rep. 8th ed., US Department of Health and Human Services (USDHHS) and US Department of Agriculture (USDA) (2015).

[27] Herforth, A. *et al.* A global review of food-based dietary guidelines. *Advances in Nutrition* **10**, 590–605 (2019).

[28] Drewnowski, A., Dwyer, J., King, J. C. & Connie M Weaver. A proposed nutrient density score that includes food groups and nutrients to better align with dietary guidance. *Nutrition Reviews* **77**, 404–416 (2019).

[29] Fulgoni, V. L., Keast, D. R. & Drewnowski, A. Development and Validation of the Nutrient-Rich Foods Index: A Tool to Measure Nutritional Quality of Foods. *The Journal of Nutrition* **139**, 1549–1554 (2009).

[30] Drewnowski, A. & Fulgoni, V. L. Nutrient density: Principles and evaluation tools. *The American Journal of Clinical Nutrition* **99**, 1223S–1228S (2014).

[31] Drewnowski, A. & Burton-Freeman, B. A new category-specific nutrient rich food (NRF9f.3) score adds flavonoids to assess nutrient density of fruit. *Food & Function* **11**, 123–130 (2020).

[32] Drewnowski, A., Gonzalez, T. D. & Rehm, C. D. Balanced Hybrid Nutrient Density Score Compared to Nutri-Score and Health Star Rating Using Receiver Operating Characteristic Curve Analyses. *Frontiers in Nutrition* **9**, 867096 (2022).

[33] Committee to Review the Dietary Reference Intakes for Sodium and Potassium, Food and Nutrition Board, Health and Medicine Division & National Academies of Sciences, Engineering, and Medicine. *Dietary Reference Intakes for Sodium and Potassium* (National Academies Press, Washington, D.C., 2019).

[34] Mainardi, F., Drewnowski, A. & Green, H. Personalized nutrient profiling of food patterns: Nestlé's nutrition algorithm applied to dietary intakes from NHANES. *Nutrients* **11**, 379 (2019).

[35] Standing Committee for the Review of the Dietary Reference Intake Framework, Food and Nutrition Board, Health and Medicine Division & National Academies of Sciences, Engineering, and Medicine. *Rethinking the Acceptable Macronutrient Distribution Range for the 21st Century: A Letter Report* (National Academies Press, Washington, D.C., 2024).

[36] World Health Organization. *WHO Global Report on Sodium Intake Reduction* 1st edn (World Health Organization, Geneva, 2023).

[37] World Health Organization. *Guideline: Sugars Intake for Adults and Children* (World Health Organization, Geneva, 2015).

[38] World Health Organization. *Saturated Fatty Acid and Trans-Fatty Acid Intake for Adults and Children: WHO Guideline* (World Health Organization, Geneva, 2023).
